# Biological Evaluation and Docking Studies of New Carbamate, Thiocarbamate, and Hydrazide Analogues of Acyl Homoserine Lactones as *Vibrio fischeri*-Quorum Sensing Modulators

**DOI:** 10.3390/biom10030455

**Published:** 2020-03-15

**Authors:** Qiang Zhang, Yves Queneau, Laurent Soulère

**Affiliations:** Univ Lyon, Université Claude Bernard Lyon 1, INSA Lyon, CPE Lyon, UMR 5246, CNRS, ICBMS, Institut de Chimie et de Biochimie Moléculaires et Supramoléculaires, Chimie Organique et Bioorganique, Bât. E. Lederer, 1 rue Victor Grignard, F-69622 Villeurbanne, France; qiang.zhang@insa-lyon.fr

**Keywords:** carbamate analogues, modulators, quorum sensing, docking

## Abstract

A series of carbamate, thiocarbamate, and hydrazide analogues of acylhomoserine lactones (AHLs) were synthesized and their ability to modulate *Vibrio fischeri*-quorum sensing was evaluated. The compounds in the series exhibit variable side chain length and the possible presence of a diversely substituted phenyl substituent. Biological evaluation on the *Vibrio fischeri* quorum sensing system revealed that the ethyl substituted carbamate (**1**) display a weak agonistic activity whereas compounds with longer chain length or benzyl substituents display significant antagonistic activity. The most active compounds in the series were the 4-nitrobenzyl carbamate and thiocarbamate **7** and **11** which exhibited an IC_50_ value of about 20 µM. These activities are in the range of other reported of AHL-structurally related quorum sensing (QS) inhibitors. Docking experiments conducted on the LuxR model showed that, compared to the natural ligand OHHL, the additional heteroatom of the carbamate group induces a new hydrogen bond with Tyr70 leading to a different global hydrogen-bond network. Tyr70 is an important residue in the binding site and is strictly conserved in the LuxR family. For the 4-nitrobenzyl carbamate and thiocarbamate analogues, the docking results highlight an additional hydrogen bond between the nitro group and Lys178. For hydrazide analogues, which are deprived of any activity, docking shows that the orientation of the carbonyl group is opposite as compared with the natural ligand, leading to the absence of a H-bond between the C=O with Tyr62. This suggests that, either this later interaction, or the influence of the C=O orientation on the overall ligand conformation, are essential for the biological activity.

## 1. Introduction

**Figure 1:**
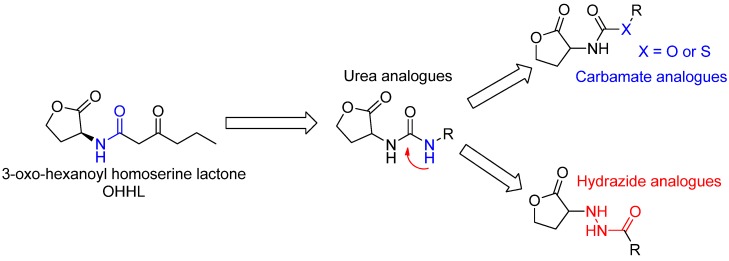
Design of analogues of OHHL modified on the amide functional group.

Acylhomoserine lactones (AHLs) are molecular signals used by Gram negative bacteria to communicate. They are biosynthesized by proteins of the LuxI family and are recognized by their cognate transcriptional regulators belonging to the LuxR family. This system referred to as quorum sensing (QS) allows bacteria to coordinate their behaviors according to their environment [1,2,3,4,5]. The production of AHLs monitors the regulation of genes encoding for different phenotypes such as biofilm formation, bioluminescence, and virulence. QS can thus be modulated by small AHLs analogues [3,6,7,8,9,10,11,12]. To date, we reported the synthesis and QS activity of several AHLs analogues in which the amide function was replaced by bioisosteric functions such as urea [13,14], sulfonamide [15], reverse amide [16], and heterocycles [17]. Carbamates and thiocarbamates have been extensively used as bioisosteric analogues of amide bioactive compounds, as mentioned by Ghosh and Brindisi [18]. Although, a comprehensive study of this type of compounds in the field of QS modulation is missing, since to our knowledge, only one example has been described in the literature with a hexyl substituted carbamate as TraR modulator [19]. Building on our work on *Vibrio fischeri*-quorum sensing modulation using urea-type AHLs analogues [13], we report here the synthesis, the biological evaluation, and docking studies of carbamate (eight compounds) and thiocarbamate (three compounds) AHL analogues for which the urea additional NH group is replaced by an oxygen or a sulfur atom (Figure 1). We also described three hydrazide analogues of AHLs for which the urea functional group has been inverted as shown in Figure 1.

## 2. Materials and Methods

### 2.1. Synthesis

All commercial materials were used without further purification. Flash chromatography was carried out using Macherey-Nagel (Hoerdt, France) Kieselgel 60 M silica. Analytical thin layer chromatography was realized using aluminum-backed plates coated with Macherey-Nagel Kieselgel 60 XtraSIL G/UV254. Compounds were visualized under UV light (at 254 nm) or stained using KMnO_4_. Nuclear magnetic resonance (NMR) spectra were recorded on a Bruker AVL300 or a Bruker AV400 or a Bruker AV500 spectrometer (Billerica, MA, USA), operating respectively at 300, 400, and 500 MHz for the proton (^1^H) NMR. Carbon (^13^C) NMR spectra were recorded on a Bruker AVL300 or a Bruker AV400 spectrophotometer or a Bruker AV500 spectrometer, operating, respectively, at 75, 100, and 125 MHz. Chemical shifts were reported in parts per million (ppm) in the scale relative to residual solvent signals. Multiplicities are abbreviated as follows: s, singlet; d, doublet; t, triplet; dd, doublet of doublets; dt, doublet of triplts, m, multiplet; br, broad. Coupling constants were measured in Hertz (Hz). High-resolution mass spectra (HRMS) and low-resolution mass spectra were performed by the Centre Commun de Spectrométrie de Masse (CCSM), University of Lyon 1, Lyon, France. l- or d-homoserine lactone hydrobromide was synthesized as previously described [20].

#### 2.1.1. General Procedure for Route A

To a solution of l-α-amino-γ-butyrolactone hydrobromide (100 mg, 0.55 mmol) in anhydrous THF (2 mL) *N*,*N*-diisopropylethylamine (0.213 mL, 0.66 mmol) at 0 °C was added, and the appropriate commercially available benzyl chloroformate or ethyl chloroformate (1.2 eq) was added dropwise. The mixture was stirred overnight while the temperature was warmed up to room temperature. The mixture was then diluted with EtOAc (10 mL) and washed with brine (10 mL). The organic phase was collected, dried over anhydrous Na_2_SO_4_, concentrated, and the residue was purified by flash chromatography to give compounds **1** and **5** as white solids.

***N*-(ethoxycarbonyl)-l-homoserine lactone** (**1**). Flash chromatography of the crude product (1:1 EtOAc-pentane) afforded **1** (72%) as a white solid. IR (cm^−1^): 3332 (N-H), 1785 (C=O, lactone), 1679 (C=O, carbamate), 1537 (-NHCO-). [α${]}_{D}^{24}$ = −43.9 (c = 0.34, acetone). NMR data were consistent with the literature [21].

***N*-(benzyloxycarbonyl)-l-homoserine lactone** (**5**). Flash chromatography of the crude product (1:3 EtOAc-pentane) afforded **5** (48%) as a white solid. IR (cm^−1^): 3324 (N-H), 1776 (C=O, lactone), 1686 (C=O, carbamate), 1539 (-NHCO-). [α${]}_{D}^{24}$ = −27.9 (c = 0.29, acetone). NMR data were consistent with the literature [22].

***N*-(4-nitrobenzylthiocarbonyl)-l-homoserine lactone** (**11**). To a solution of triphosgene (1 mmol) in DCM (2 mL) 4-nitro benzylmercaptan (2.4 mmol) was added slowly which was dissolved in DCM (2 mL) at 0 °C, followed by addition of pyridine (0.2 mL, 2.48 mmol) dissolved in DCM (2 mL). After stirring for 2 h at 0 °C, the reaction was quenched by addition of water (2 mL). The reaction mixture was diluted with DCM (16 mL) and washed with ice-cold water (8 mL). The organic layer was dried over anhydrous Na_2_SO_4_, concentrated to afford the S-4-nitrobenzyl chlorothioformate (390 mg, yield 70%) as a colorless oil, used immediately without further purification. ^1^H NMR (300 MHz, Chloroform-*d*) δ 8.21 (d, *J* = 8.9 Hz, 2H, Ph), 7.51 (d, *J* = 8.7 Hz, 2H, Ph), 4.22 (s, 2H, CH_2_). Following the general procedure for the route **A**, flash chromatography of the crude product (1:1 EtOAc-pentane) afforded **11** (51%) as a white solid. IR (cm^−1^): 3307 (N-H), 1782 (C=O, lactone), 1640 (C=O, thiocarbamate), 1513 (-NHCO-), 1491 (Ar-NO_2_), 1345 (Ar-NO_2_). [α${]}_{D}^{24}$ = −30.4 (c = 0.09, acetone). ^1^H NMR (300 MHz, Chloroform-*d*) δ 8.15 (d, *J* = 8.8 Hz, 2H, Ph), 7.49 (d, *J* = 8.7 Hz, 2H, Ph), 6.24 (d, *J* = 6.0 Hz, 1H, N**H**), 4.57 (m, 1H, C**H**), 4.46 (m, 1H, OC***H***H-lactone), 4.27 (m, 1H, OC***H***H-lactone), 4.19 (s, 2H, SCH_2_), 2.79 (m, 1H, C***H***H-lactone), 2.22 (m, 1H, C***H***H-lactone). ^13^C NMR (75 MHz, Chloroform-*d*) δ 174.8 (CO-lactone), 167.1 (S**C**ONH), 147.1 (Ph), 145.8 (Ph), 129.7 (2C, Ph), 123.8 (2C, Ph), 66.0 (O**C**H_2_-lactone), 50.8 (**C**H-NH), 33.4 (S**C**H_2_), 30.1 (**C**H_2_-lactone). ESI-HRMS (M + Na)^+^: 319.0359; found: 319.0355.

#### 2.1.2. General Procedure for Route B

Carbamates: (i) To a stirred solution of different alcohols (1.05 eq) in THF, triethylamine (2.3 eq) and *p*-nitrophenyl chloroformate (1 eq) at 0 °C were added. The reaction mixture was allowed to warm up to room temperature and was stirred overnight. The mixture was then diluted with EtOAc (20 mL) and filtered. The solution was washed with brine (2 × 10 mL); the organic phase was collected, dried over anhydrous Na_2_SO_4_, concentrated to afford the desired carbonates. (ii) To a solution of l-α-amino-γ-butyrolactone hydrobromide (1 eq) in dry THF (2 mL), triethylamine (1 eq) and various *p*-nitrophenyl carbonates (1.2 eq) dissolved in THF (1 mL) were added at room temperature. The reaction mixture was stirred at 40 °C overnight. The solution was filtered and the solvent was evaporated. The residue was purified by flash chromatography to give the corresponding carbamates as white solids.

Thiocarbamates: (i) To a solution of different thiols (1 eq) in dry DCM, triethylamine (1.05 eq), 4-dimethylaminopyridine (DMAP) (1.05 eq), and *p*-nitrophenyl chloroformate (1.05 eq) at 0 °C were added, respectively. The reaction mixture was allowed to warm up to room temperature and stirred overnight. The mixture was diluted with DCM (20 mL) and washed with water (2 × 10 mL), the organic phase was collected, dried over anhydrous Na_2_SO_4_, concentrated, and purified by flash chromatography to get the desired thiocarbonate. (ii) To a solution of l-α-amino-γ-butyrolactone hydrobromide (1 eq) in dry THF (2 mL), triethylamine (1 eq or 1.2 eq) and *p*-nitrophenyl thiocarbonates (1.2 eq) dissolved in THF (1 mL) at room temperature were added. The reaction mixture was then stirred at 60 °C overnight. The reaction mixture was cooled to room temperature, filtered, and the solvent was evaporated. The residue was purified by flash chromatography to give the corresponding thiocarbamates as white solids.

***N*-(butoxycarbonyl)-l-homoserine lactone** (**2**). From butyl alcohol the Butyl 4-nitrophenyl carbonate was obtained (48%) as a yellow liquid. ^1^H NMR (300 MHz, Chloroform-*d*) δ 8.18 (d, *J* = 9.2 Hz, 2H, Ph), 7.30 (d, *J* = 9.2 Hz, 2H, Ph), 4.21 (t, *J* = 6.6 Hz, 2H, OC**H_2_**), 1.80–1.56 (m, 2H, C**H_2_**), 1.44–1.26 (m, 2H, C**H_2_**), 0.89 (t, *J* = 7.4 Hz, 3H, C**H_3_**). According to the general procedure B, flash chromatography of the crude product (1:2 EtOAc-pentane) afforded **2** (68%) as a white solid. IR (cm^−1^): 3333 (N-H), 1775 (C=O, lactone), 1688 (C=O, carbamate), 1537 (-NHCO-). [α${]}_{D}^{24}$ = −30 (c = 0.44, acetone). ^1^H NMR (500 MHz, Chloroform-*d*) δ 5.39 (s, 1H, N**H**), 4.56–4.33 (m, 2H, OC***H***H-lactone, C**H**), 4.24 (m, 1H, OC***H***H-lactone), 4.07 (t, *J* = 6.7 Hz, 2H, OC**H_2_**), 2.73 (m, 1H, C***H***H-lactone), 2.21 (m, 1H, C***H***H-lactone), 1.59 (m, 2H, C**H_2_**), 1.36 (m, 2H, C**H_2_**), 0.91 (t, *J* = 7.4 Hz, 3H, C**H_3_**). ^13^C NMR (126 MHz, Chloroform-*d*) δ 175.2 (**C**O-lactone), 156.5 (O**C**ONH), 65.8 (O**C**H_2_-lactone), 65.5 (O**C**H_2_), 50.4 (**C**H-NH), 30.9 (**C**H_2_), 30.3 (**C**H_2_-lactone), 19.0 (**C**H_2_), 13.7 (**C**H_3_). ESI-HRMS (M + Na)^+^: 224.0893; found: 224.0892.

**N-(butoxycarbonyl)-d-homoserine lactone** (**3**). Compound **3** was obtained from Butyl 4-nitrophenyl carbonate and d-α-amino-γ-butyrolactone hydrobromide. Flash chromatography of the crude product (1:3 EtOAc-pentane) afforded **3** (74%) as a white solid. IR (cm^−1^): 3329 (N-H), 1775 (C=O, lactone), 1687 (C=O, carbamate), 1536 (-NHCO-). [α${]}_{D}^{24}$ = +35.3 (c = 0.4, acetone). ^1^H NMR (500 MHz, Chloroform-*d*) δ 5.49 (d, *J* = 6.5 Hz, 1H, N**H**), 4.40 (m, 2H, OC***H***H-lactone, C**H**), 4.22 (m, 1H, OC***H***H-lactone), 4.05 (t, *J* = 6.8 Hz, 2H, OC**H_2_**), 2.69 (m, 1H, C***H***H-lactone), 2.21 (m, 1H, C***H***H-lactone), 1.57 (m, 2H, C**H_2_**), 1.40–1.24 (m, 2H, C**H_2_**), 0.94–0.84 (t, *J* = 7.3 Hz, 3H, C**H_3_**). ^13^C NMR (126 MHz, Chloroform-*d*) δ 175.4 (**C**O-lactone), 156.6 (O**C**ONH), 65.8 (O**C**H_2_-lactone), 65.5 (O**C**H_2_), 50.4 (**C**H-NH), 31.0 (**C**H_2_), 30.1 (**C**H_2_-lactone), 19.1 (**C**H_2_), 13.7 (**C**H_3_). ESI-HRMS (M + Na)^+^: 224.0893; found: 224.0897.

**N-(hexyloxycarbonyl)-l-homoserine lactone** (**4**). From hexyl alcohol Hexyl 4-nitrophenyl carbonate was obtained (56%) as a yellow liquid. ^1^H NMR (300 MHz, Chloroform-*d*) δ 8.25 (d, *J* = 9.2 Hz, 2H, Ph), 7.36 (d, *J* = 9.2 Hz, 2H, Ph), 4.27 (t, *J* = 6.7 Hz, 2H, OC**H_2_**), 1.83–1.61 (m, 2H, C**H_2_**), 1.51–1.19 (m, 6H, 3 × C**H_2_**), 0.89 (t, *J* = 6.6 Hz, 3H, C**H_3_**). According to the general procedure B, flash chromatography of the crude product (1:2 EtOAc-pentane) afforded **4** (63%) as a white solid. IR (cm^−1^): 3332 (N-H), 1775 (C=O, lactone), 1688 (C=O, carbamate), 1539 (-NHCO-). [α${]}_{D}^{24}$ = −26.9 (c = 0.35, acetone). ^1^H NMR (500 MHz, Chloroform-*d*) δ 5.40 (s, 1H, N**H**), 4.41 (m, 2H, OC***H***H-lactone, C**H**), 4.24 (m, 1H, OC***H***H-lactone), 4.05 (t, *J* = 6.7 Hz, 2H, OC**H_2_**), 2.72 (m, 1H, C***H***H-lactone), 2.21 (m, 1H, C***H***H-lactone), 1.59 (m, 2H, C**H_2_**), 1.43–1.19 (m, 6H, C**H_2_**C**H_2_**C**H_2_**), 0.86 (t, *J* = 6.6 Hz, 3H, C**H_3_**). ^13^C NMR (126 MHz, Chloroform-*d*) δ 175.2 (**C**O-lactone), 156.5 (O**C**ONH), 65.8 (O**C**H_2_-lactone), 65.8 (O**C**H_2_), 50.4 (**C**H-NH), 31.4 (**C**H_2_), 30.3 (**C**H_2_-lactone), 28.8 (**C**H_2_), 25.5 (**C**H_2_), 22.5 (**C**H_2_), 14.0 (**C**H_3_). ESI-HRMS (M + Na)^+^: 252.1206; found: 252.1207.

***N*-(4-bromobenzyloxycarbonyl)-**l**-homoserine lactone** (**6**). From 4-bromobenzyl alcohol (4-Bromoophenyl)methyl 4-nitrophenyl carbonate was obtained by flash chromatography of the crude product (1:8 EtOAc-pentane) in 47% yield as a white solid. IR (cm^−1^): 3306 (N-H), 1776 (C=O, lactone), 1695 (C=O, carbamate), 1538 (-NHCO-). [α${]}_{D}^{24}$ = −23.5 (c = 0.19, acetone). ^1^H NMR (300 MHz, Chloroform-*d*) δ 8.27 (d, *J* = 9.2 Hz, 2H, Ph), 7.54 (d, *J* = 8.4 Hz, 2H, Ph), 7.43–7.28 (m, 4H, Ph), 5.24 (s, 2H, OC**H_2_**Ar). ^13^C NMR (76 MHz, Chloroform-*d*) δ 155.4 (O**C**O), 152.4 (Ph), 145.5 (Ph), 133.2 (Ph), 132.0 (2C, Ph), 130.3 (2C, Ph), 125.3 (2C, Ph), 123.3 (Ph), 121.8 (2C, Ph), 70.1 (O**C**H_2_Ar). ESI-HRMS (M + Na)^+^: 373.9635; found: 373.9637. According to the general procedure B, flash chromatography of the crude product (1:3 EtOAc-pentane) afforded **6** (40%) as a white solid. ^1^H NMR (400 MHz, Chloroform-*d*) δ 7.48 (d, *J* = 8.4 Hz, 2H, Ph), 7.22 (d, *J* = 8.4 Hz, 2H, Ph), 5.39 (s, 1H, N**H**), 5.07 (s, 2H, OC**H_2_**Ar), 4.42 (m, 2H, OC***H***H-lactone, C**H**), 4.25 (m, 1H, OC***H***H-lactone), 2.88–2.66 (m, 1H, C***H***H-lactone), 2.21 (m, 1H, C***H***H-lactone). ^13^C NMR (101 MHz, Chloroform-*d*) δ 174.9 (**C**O-lactone), 156.0 (O**C**ONH), 134.9 (Ph), 131.7 (2C, Ph), 129.8 (2C, Ph), 122.37 (Ph), 66.5 (O**C**H_2_Ar), 65.8 (O**C**H_2_-lactone), 50.5 (**C**H-NH), 30.4 (**C**H_2_-lactone). ESI-HRMS (M + Na)^+^: 335.9842; found: 335.9843.

***N*-(4-nitrobenzyloxycarbonyl)-l-homoserine lactone** (**7**). From 4-nitrobenzyl alcohol (4-Nitrophenyl)methyl 4-nitrophenyl carbonate was obtained in 78% yield as a yellow solid. ^1^H NMR data were consistent with the literature [23]. According to the general procedure B, flash chromatography of the crude product (1:1 EtOAc-pentane) afforded **7** (52%) as a white solid. IR (cm^−1^): 3339 (N-H), 1783 (C=O, lactone), 1692 (C=O, carbamate), 1533 (-NHCO-), 1512 (Ar-NO_2_), 1345 (Ar-NO_2_). [α${]}_{D}^{24}$ = −24.5 (c = 0.45, acetone). NMR data were consistent with the literature [24].

***N*-(4-phenylbutoxycarbonyl)-l-homoserine lactone** (**8**). From 4-phenyl butanol, flash chromatography of the crude product (1:8 EtOAc-pentane) afforded (4-Phenyl)butyl 4-nitrophenyl carbonate (48%) as a white solid. ^1^H NMR (300 MHz, Chloroform-*d*) δ 8.24 (d, *J* = 8.4 Hz, 2H, Ph), 7.34 (d, *J* = 8.4 Hz, 2H, Ph), 7.32–7.11 (m, 5H, Ph), 4.29 (t, *J* = 6.0 Hz, 2H, OC**H_2_**), 2.67 (t, *J* = 6.9 Hz, 2H, C**H_2_**), 1.94–1.68 (m, 4H, C**H_2_**C**H_2_**). ^13^C NMR (76 MHz, Chloroform-*d*) δ 155.6 (O**C**O), 152.6 (Ph), 145.4 (Ph), 141.7 (Ph), 128.5 (2C, Ph), 128.4 (2C, Ph), 126.0 (Ph), 125.3 (2C, Ph), 121.9 (2C, Ph), 69.4 (O**C**H_2_), 35.4 (**C**H_2_), 28.1 (**C**H_2_), 27.5 (**C**H_2_). ESI-HRMS (M + Na)^+^: 338.0999; found: 338.0999. Following general procedure B, flash chromatography of the crude product (1:2 EtOAc-pentane) afforded **8** (40%) as a white solid. IR (cm^−1^): 3336 (N-H), 1776 (C=O, lactone), 1687 (C=O, carbamate), 1537 (-NHCO-). [α${]}_{D}^{24}$ = −17.4 (c = 0.13, acetone). ^1^H NMR (500 MHz, Chloroform-*d*) δ 7.33–7.23 (m, 2H, Ph), 7.22–7.12 (m, 3H, Ph), 5.25 (d, *J* = 12.3 Hz, 1H, N**H**), 4.41 (m, OC***H***H-lactone, C**H**), 4.25 (m, 1H, OC***H***H-lactone), 4.12 (t, *J* = 6.2 Hz, 2H, OC**H_2_**), 2.77 (m, 1H, C***H***H-lactone), 2.64 (t, *J* = 7.1 Hz, 2H, C**H_2_**Ph), 2.20 (m, 1H, C***H***H-lactone), 1.69 (m, 4H, C**H_2_**C**H_2_**). ^13^C NMR (126 MHz, CDCl_3_) δ 175.1 (**C**O-lactone), 156.4 (O**C**ONH), 142.1 (Ph), 128.5 (2C, Ph), 128.4 (2C, Ph), 125.9 (Ph), 65.9 (O**C**H_2_-lactone), 65.6 (O**C**H_2_), 50.5 (**C**H-NH), 35.5 (**C**H_2_), 30.6 (**C**H_2_-lactone), 28.5 (**C**H_2_), 27.7 (**C**H_2_). ESI-HRMS (M + Na)^+^: 300.1206; found: 300.1210.

***N*-(butylthiocarbonyl)-l-homoserine lactone** (**9**). From butyl mercaptan, flash chromatography of the crude product (1:10 EtOAc-pentane) afforded Butyl 4-nitrophenyl thiocarbonate (62%) as a light yellow liquid. ^1^H NMR (300 MHz, Chloroform-*d*) δ 8.27 (d, *J* = 9.2 Hz, 2H, Ph), 7.35 (d, *J* = 9.2 Hz, 2H, Ph), 2.96 (t, *J* = 7.3 Hz, 2H, SC**H_2_**), 1.82–1.63 (m, 2H, C**H_2_**), 1.53–1.33 (m, 2H, C**H_2_**), 0.95 (t, *J* = 7.3 Hz, 3H, C**H_3_**). ^13^C NMR (76 MHz, Chloroform-*d*) δ 169.77 (S**C**O), 155.65 (Ph), 145.33 (Ph), 125.29 (2C, Ph), 122.06 (2C, Ph), 31.46 (**C**H_2_), 31.20(**C**H_2_), 21.81 (**C**H_2_), 13.54 (**C**H_3_). ESI-HRMS (M + Na)^+^: 278.0457; found: 278.0462. Following general procedure B, flash chromatography of the crude product (1:3 EtOAc-pentane) afforded **9** (46%) as a white solid. IR (cm^−^^1^): 3315 (N-H), 1775 (C=O, lactone), 1643 (C=O, thiocarbamate), 1522 (-NHCO-). [α${]}_{D}^{24}$ = −13.4 (c = 0.17, acetone). ^1^H NMR (300 MHz, Chloroform-*d*) δ 6.12 (d, *J* = 5.9 Hz, 1H, N**H**), 4.56 (m, 1H, C**H**), 4.50–4.37 (m, 1H, OC***H***H-lactone), 4.27 (m, 1H, OC***H***H-lactone), 2.91 (t, *J* = 7.3 Hz, 2H, SC**H_2_**), 2.80 (m, 1H, C***H***H-lactone), 2.22 (m, 1H, C***H***H-lactone), 1.61 (m, 2H, C**H_2_**), 1.39 (m, 2H, C**H_2_**), 0.91 (t, *J* = 7.3 Hz, 3H, C**H_3_**). ^13^C NMR (76 MHz, Chloroform-*d*) δ 175.0 (**C**O-lactone), 168.9 (S**C**ONH), 66.0 (O**C**H_2_-lactone), 50.6 (**C**H-NH), 32.2 (**C**H_2_), 30.4 (**C**H_2_-lactone), 29.8 (S**C**H_2_), 21.9 (**C**H_2_), 13.6 (**C**H_3_). ESI-HRMS (M + Na)^+^: 240.0664; found: 240.0653.

***N*-(benzylthiocarbonyl)-l-homoserine lactone** (**10**). From benzyl mercaptan, flash chromatography of the crude product (1:11 EtOAc-pentane) afforded Benzyl 4-nitrophenyl thiocarbonate (39%) as a white solid. ^1^H NMR (300 MHz, Chloroform-*d*) δ 8.27 (d, *J* = 9.2 Hz, 2H, Ph), 7.43–7.27 (m, 7H, Ph), 4.20 (s, 2H, SC**H_2_**). ^13^C NMR (76 MHz, Chloroform-*d*) δ 169.24 (S**C**O), 155.57 (Ph), 145.43 (Ph), 136.15 (Ph), 128.97 (2C, Ph), 128.85 (2C, Ph), 127.90 (Ph), 125.32 (2C, Ph), 122.07 (2C, Ph), 35.84 (S**C**H_2_Ph). ESI-HRMS (M + Na)^+^: 312.0301; found: 312.0302. Following general procedure B, flash chromatography of the crude product (1:3 EtOAc-pentane) afforded **10** (27%) as a white solid. IR (cm^−1^): 3321 (N-H), 1776 (C=O, lactone), 1661 (C=O, thiocarbamate), 1518 (-NHCO-). [α${]}_{D}^{24}$ = −12.6 (c = 0.11, acetone). ^1^H NMR (500 MHz, Chloroform-*d*) δ 7.35–7.26 (m, 4H, Ph), 7.26–7.21 (m, 1H, Ph), 6.12 (s, 1H, N**H**), 4.55 (m, 1H, C**H**), 4.44 (m, 1H, OC***H***H-lactone), 4.25 (m, 1H, OC***H***H-lactone), 4.17 (s, 2H, SC**H_2_**), 2.79 (m, 1H, C***H***H-lactone), 2.21 (m, 1H, C***H***H-lactone). ^13^C NMR (126 MHz, Chloroform-*d*) δ 174.8 (**C**O-lactone), 168.1 (S**C**ONH), 137.6 (Ph), 128.8 (2C, Ph), 128.6 (2C, Ph), 127.4 (Ph), 66.0 (O**C**H_2_-lactone), 50.7 (**C**H-NH), 34.3 (S**C**H_2_Ph), 30.4 (**C**H_2_-lactone). ESI-HRMS (M + Na)^+^: 274.0508; found: 274.0511.

#### 2.1.3. General Procedure for Route C (Hydrazides)

(i) To a 25 mL flask, 4 mL of hydrazine (80% in water) and carboxylic esters (1 g) were added. The mixture was stirred at 70 °C for 45 min and then cooled to room temperature. The reaction mixture was then extracted with EtOAc (30 mL) and washed with brine (30 mL). The organic phase was dried over anhydrous Na_2_SO_4_, concentrated, and the residue was purified by flash chromatography to give corresponding hydrazide compounds.

(ii) To a solution of different hydrazides (1.0 eq) in dry DCM, DIEA (2.0 eq), α-Bromo-γ-butyrolactone (2.0 eq), and TBABr (0.3 eq) were added, respectively. The reaction mixture was stirred at 40 °C overnight. The solvent was evaporated. The residue was purified by flash chromatography to give the corresponding hydrazide AHL analogues.

**N’-(2-oxotetrahydrofuran-3-yl)butyrohydrazide** (**12**). From methyl butanoic ester, Butyrohydrazide was obtained by flash chromatography of the crude product (95:5 EtOAc-MeOH) in 90% yield. NMR data were consistent with the literature [25]. According to the general procedure C, flash chromatography of the crude product (EtOAc) afforded **12** (82%). IR (cm^−1^): 3299 (N-H), 1774 (C=O, lactone), 1637 (C=O, hydrazide), 1538 (-NHCO-). ^1^H NMR (300 MHz, Chloroform-*d*) δ 7.60 (s, 1H, N**H**), 4.40 (m, 1H, OC***H***H- lactone), 4.21 (m, 1H, OC***H***H-lactone), 4.12 (br, 1H, NH), 3.91 (m, 1H, C**H**-NH), 2.48 (m, 1H, C***H***H-lactone), 2.29–2.08 (m, 3H, C***H***H-lactone, C**H_2_**CO), 1.68 (m, 2H, C**H_2_**-CH_3_), 0.94 (t, *J* = 7.4 Hz, 3H, C**H_3_**). ^13^C NMR (101 MHz, Chloroform-*d*) δ 176.0 (**C**O), 173.2 (**C**O), 65.8 (O**C**H_2_-lactone), 57.9 (**C**H-NH), 36.3 (**C**H_2_CO), 27.5 (**C**H_2_-lactone), 18.9 (**C**H_2_CH_3_), 13.7 (**C**H_3_). ESI-HRMS (M + Na)^+^: 209.0897; found: 209.0900.

**N’-(2-oxotetrahydrofuran-3-yl)hexanehydrazide** (**13**). From methyl hexanoic ester Hexanehydrazide was obtained by flash chromatography of the crude product (95:5 EtOAc-MeOH) in 74% yield. NMR data were consistent with the literature [24]. According to the general procedure C, flash chromatography of the crude product (4:1 EtOAc-pentane) afforded **13** (48%). IR (cm^−1^): 3326 (N-H), 1762 (C=O, lactone), 1641 (C=O, hydrazide), 1525 (-NHCO-). ^1^H NMR (300 MHz, Chloroform-*d*) δ 7.56 (s, 1H, N**H**), 4.41 (m, 1H, OC***H***H-lactone), 4.22 (m, 1H, OC***H***H-lactone), 3.93 (m, 1H, C**H**-NH), 3.84 (br, 1H, N**H**), 2.49 (m, 1H, C***H***H-lactone), 2.33–2.09 (m, 3H, C***H***H-lactone, C**H_2_**CO), 1.62 (m, 2H, C**H_2_**CH**_2_**CO), 1.41–1.18 (m, 4H, C**H_2_**C**H_2_**CH_3_), 0.97–0.78 (m, 3H, C**H_3_**). ^13^C NMR (101 MHz, Chloroform-*d*) δ 175.9 (**C**O), 173.3 (**C**O), 65.8 (O**C**H_2_-lactone), 57.9 (**C**H-NH), 34.5 (**C**H_2_CO), 31.4 (**C**H_2_CH_2_CH_3_), 27.5 (**C**H_2_-lactone), 25.1 (**C**H_2_CH_2_CH_2_CH_3_), 22.3 (**C**H_2_CH_3_), 13.9 (**C**H_3_). ESI-HRMS (M + H)^+^: 215.1390; found: 215.1394.

**N’-(2-oxotetrahydrofuran-3-yl)-4-phenylbutanehydrazide** (**14**). From ethyl 4-phenylbutanoic ester, 4-Phenylbutanehydrazide was obtained as a white solid (96%). NMR data were consistent with the literature [26]. According to the general procedure C, flash chromatography of the crude product (EtOAc) afforded **14** (63%). IR (cm^−1^): 3235 (N-H), 1769 (C=O, lactone), 1640 (C=O, hydrazide), 1539 (-NHCO-). ^1^H NMR (400 MHz, Chloroform-*d*) δ 7.57 (s, 1H, N**H**), 7.32–7.26 (m, 2H, Ph), 7.22–7.12 (m, 3H, Ph), 4.39 (m, 1H, OC***H***H- lactone), 4.20 (m, 1H, OC***H***H-lactone), 3.89 (m, 1H, C**H**-NH), 3.78 (br, 1H, N**H**), 2.65 (t, *J* = 7.5 Hz, 2H, C**H_2_**CO), 2.47 (m, 1H, C***H***H-lactone), 2.27–2.13 (m, 3H, C***H***H-lactone, C**H_2_**Ph), 2.06–1.88 (m, 2H, C**H_2_**CH_2_Ph). ^13^C NMR (101 MHz, Chloroform-*d*) δ 175.8 (**C**O), 172.8 (**C**O), 141.1 (Ph), 128.5 (2C, Ph), 128.5 (2C, Ph), 126.1 (Ph), 65.8 (O**C**H_2_-lactone), 57.9 (**C**H-NH), 35.1 (**C**H_2_CO), 33.7 (**C**H_2_Ph), 27.5 (**C**H_2_-lactone), 26.8 (**C**H_2_CH_2_Ph). ESI-HRMS (M + Na)^+^: 285.1210; found: 285.1203.

### 2.2. Biological Evaluation

The recombinant *Escherichia coli* strain NM522 with the sensor plasmid pSB401 was used to measure the induction of luminescence. Bacterial cultures were grown in Luria broth (50 mL) at 30 °C. After 18 h, this bacterial culture was diluted 10 times to be used in the assay using 96-well plates (100 µL per well). For agonistic activity, compounds were tested at increasing concentrations in DMSO (max 2% in a total volume of 200 µL of LB). For antagonistic activity, compounds were tested in competition with OHHL (200 nM) at increasing concentrations in DMSO (max 2% in a total volume of 200 µL). This 200 nM concentration of OHHL is fully pertinent since concentrations as low as 10 or 50 nM are already valid protocols as reported in meaningful studies [27,28]. Bioluminescence was measured with a Tecan spark luminometer (Männedorf, Switzerland).

### 2.3. Molecular Modeling

Tridimensional structures of compounds were obtained from Chem3D Pro (PerkinElmer, Waltham, MA, USA). The luxR model [29] complexed with OHHL described in a previous study was used for docking studies [30]. The binding site was created by selecting residues in the neighborhood of OHHL [31]. A Docking box 15 × 15 × 15 Å center on OHHL was used for docking experiments performed with the genetical algorithm [32] docking engine of the ArgusLab software [33] with default parameters. Hydrogen bonds were assigned with a distance inferior or equal to 3 Å. Figure 3 was created using PyMOL.

## 3. Results and Discussion

### 3.1. Synthesis

**Scheme 1:**
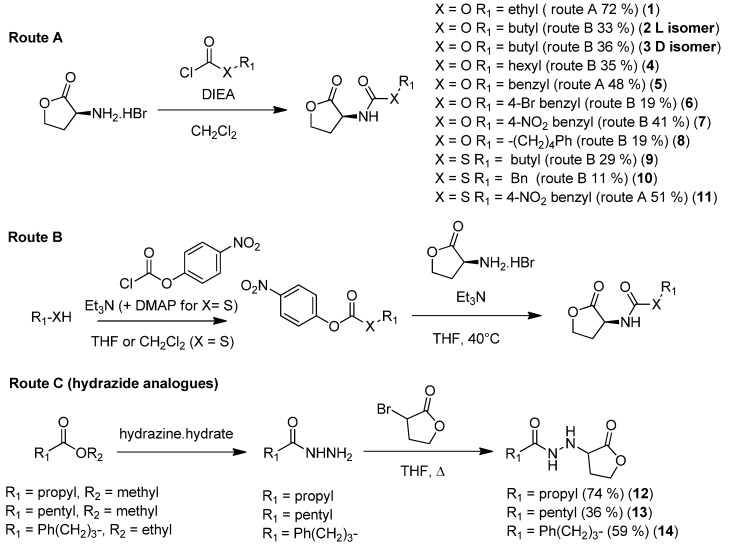
Synthesis of carbamate, thiocarbamate, and hydrazide analogues of acylhomoserine lactone.

The synthesis of ethyl (**1**) and benzyl (**5**) carbamate analogues (Scheme 1) was achieved using the corresponding commercially available ethyl or benzyl chloroformate in 72% and 48% yield, respectively (route A). For compound **11**, the S-4-nitrobenzyl chlorothioformate was first prepared using triphosgene and was then reacted with l-homoserine lactone (route A). In other cases, *para*-nitrophenyl chloroformate was first reacted with various alcohols and thiols to produce activated *para*-nitrophenyl carbonates or thiocarbonates which were further involved in reaction with l-homoserine lactone or (d-homoserine lactone for compound **3**) (route B) [34]. Thus, compounds **2**–**4** and **6**–**10** were synthesized in two steps with overall yields ranging from 11% to 41%. For hydrazide analogues, the reaction of hydrazine with various esters gave the corresponding hydrazides which were used in a nucleophilic substitution reaction with α-bromo-γ-butyrolactone furnishing hydrazides AHLs analogues **12**–**14** in good yield over two steps.

### 3.2. Biological Evaluation

All compounds were evaluated for the ability to induce bioluminescence (agonistic activity) or to decrease bioluminescence in the presence of 3-oxo-hexanoyl-l-homoserine lactone (OHHL) at 200 nM (antagonistic activity) [35]. Hydrazide AHL analogues were deprived of any activity. Among carbamate analogues, only the ethyl carbamate derivative (**1**) exhibited very weak agonistic activity by inducing at 200 µM 18% of the bioluminescence obtained with the natural ligand OHHL at a concentration of 200 nM.

**Figure 2:**
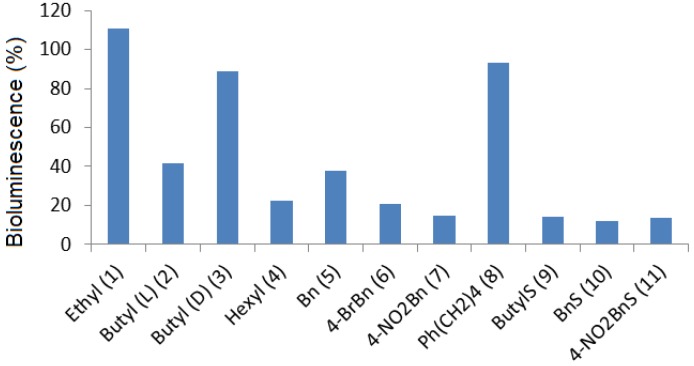
Antagonistic activity of carbamate and thiocarbamate analogues **1**–**11**: residual bioluminescence observed for all compounds at 100 µM. The luminescence was induced by OHHL at a concentration of 200 nM.

A first assessment of the antagonistic activity was performed by measuring residual bioluminescence at 100 µM concentration of analogues in competition with 200 nM of OHHL. Compounds **2**, **4**–**7**, and **9**–**11** were found to considerably decrease the bioluminescence (Figure 2). A residual bioluminescence of 42% for the l-butyl carbamate derivative **2** vs. 89% for the d-isomer **3** was measured showing the influence of the configuration on luminescence and therefore on QS inhibition [36]. A remaining luminescence of 22% was observed for the hexyl carbamate analogue **4**, the most active among alkyl carbamate analogues. With respect to aromatic analogues, **7**, **9**, **10**, and **11** were found to be the most active with a residual bioluminescence of about 10%. Interestingly, the thiocarbamates analogues **9** and **10** were found more active than the corresponding oxygenated carbamate analogues **2** and **5**.

To determine IC_50_ values, the decrease of bioluminescence of was measured using increasing concentration of each compound in presence of 200 nM of OHHL (Table 1). Values ranging from 22 to 94 µM, in the range of other reported of AHL-structurally related QS inhibitors [3,7,10], were measured for compounds **2**, **4**–**7**, and **9**–**11**. Differences in the calculated LogP values are consistent with inhibitory activity for most compounds except 4-nitrobenzyl analogues. This is the case for all thiocarbamates vs. carbamates, and also when comparing the butyl (**2**) and hexyl (**4**) derivatives, or the benzyl and 4-bromobenzyl substituents.

The 4-nitrobenzyl derivatives **7** and **11** were identified as the most active compounds in the series with and IC_50_ value of 22–23 µM. However, no additive effect of the presence of the nitro group and of the sulfur atom in the thiocarbamate was observed, although LogP of the 4-nitrobenzyl thiocarbamate is higher than the 4-nitrobenzyl carbamate like for other compounds in the series. For these two compounds, the presence of the 4-nitro substituent is therefore the key point.

### 3.3. Docking Studies

**Figure 3:**
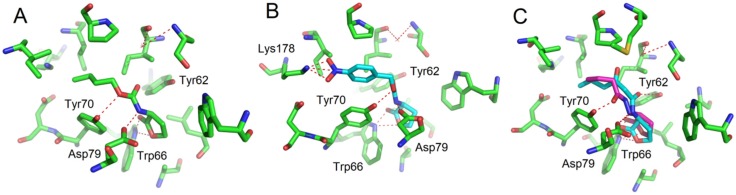
Proposed binding modes of compounds **2** (**A**) and **7** (**B**) within the binding site of the LuxR model obtained as a result of docking experiments. (**C**) Proposed binding modes of compound **12** (magenta) and OHHL (cyan) [30] obtained as a result of docking experiments. Hydrogen bonds are indicated in red dashes. Design of analogues of OHHL modified on the amide functional group.

In order to gain insight into structure–activity relationships, we performed docking studies for the active compounds using the LuxR model and a genetic algorithm. All compounds were docked as flexible ligands to study their binding modes which are described in the Table 2. Based on the results, all compounds exhibit hydrogens bonds with Trp66 and Asp 79 like the natural ligand OHHL does. For the agonist **1** bearing a small ethyl substituent, a particularly high level of flexibility was observed, leading to a binding mode comparable with the natural ligand in terms of hydrogen bonds network, however with some differences in terms of distances. For longer chain lengths (more hydrophobic compounds **2**, **4**, **9**), the alkyl chain together with the supplementary oxygen atom of the carbamate function orientate the carbamoyl group towards Tyr70 leading to hydrophobic interactions. A supplementary hydrogen bond with this amino acid is also observed (Figure 3A). The consequence of this particular orientation is that the hydrogen bond which normally exists between the C=O of the AHL amide with Tyr62 is not present, due to a distance superior to 3.5 Å between the C=O of the carbamate and Tyr62. For compounds bearing an aromatic substituent, the binding mode is globally the same, with attractive interactions and a hydrogen bond with Tyr70. For thiocarbamate derivatives, a difference with their oxygenated homologs was observed with the additional hydrogen bond with Tyr70 [37] but also with a hydrogen bond between the C=O of the carbamate group and Tyr62 as for the natural ligand. This interaction, which is not observed for carbamates, may explain the increased activity of thiocarbamate derivatives (except for nitrated compounds). Overall, an additional hydrogen bond with the Tyr70 residue is found for most carbamate/thiocarbamate analogues. The Tyr70 residue, which is a conserved residue in the LuxR proteins family (equivalent to Tyr64 in LasR [38]) is confirmed as particularly important for interactions with AHLs analogues.

For nitro compounds **7** and **11**, two additional hydrogen bonds involving the nitro group and the Lys178 residue have been identified (Figure 3B). As these two compounds are the most active in the series, these new interactions appear as a key element favoring their binding and determinant to the inhibitory activity, though with no additive effect due to the presence of the sulfur atom.

For hydrazide analogues, all inactive, docking studies revealed that the carbonyl of the hydrazide group is involved in a hydrogen bond with Tyr70 but misses the interaction with Tyr62. Indeed, the C=O is located opposite to where it is usually for natural ligands or other active analogues (Figure 3C).

Figure 3 compares the binding modes of the active carbamate **2**, the nitro compound **7** (the most active compound in the study), and the hydrazide **12** within the binding site of the LuxR model.

## 4. Conclusions

A novel family of carbamate/thiocarbamate analogues of AHLs was studied as quorum sensing modulators on the *Vibrio fisheri* system. Both agonistic and antagonistic activities were found depending on chain length and substitution. The binding mode within the LuxR binding site unveils a new hydrogen bond between the supplementary oxygen or sulfur atoms of all compounds with Tyr70, an important conserved residue in the LuxR family. The most active compounds are the *p*-nitro benzyl derivatives that exhibit additional interactions with Lys178. This study showed the ability of the carbamate/thiocarbamate functions to mimic the amide group of AHLs for fine-tuning QS modulation.

H NMR and 13C NMR data are available in Supplementary Materials.

## Figures and Tables

**Table 1:** Predicted logP and IC_50_ values for compounds **2**, **4**–**7,** and **9**–**11**. IC_50_ values were estimated with an increasing concentration of each compound in competition with 200 nM of OHHL.

Compounds	Predicted logP	IC_50_ (µM)
**Butyl (2)**	0.65	94 (±1)
**Hexyl (4)**	1.19	73 (±3)
**Bn (5)**	1.14	86 (±3)
**4-BrBn (6)**	1.97	64 (±4)
**4-NO_2_Bn (7)**	0.51	22 (±3)
**ButylS (9)**	1.22	45 (±7)
**BnS (10)**	1.71	64 (±3)
**4-NO_2_BnS (11)**	1.17	23 (±4)

**Table 2:** Occurrence and distances for hydrogen bonds between Trp66, Asp79, Tyr 62, Tyr70, and the main chemical functions of studied compounds with distances (Å). + indicates a possible H-bond; the function implicated is indicated in brackets.

Compounds	Trp66	Asp79	Tyr62	Tyr70	Lys178
OHHL (natural ligand)	+2.34 (C=O lactone)	+3.01 (NH amide)	+3.01 (C=O amide)	-	-
Ethyl (**1** agonist)	+2.44 (C=O lactone)	+3.0 (NH carbamate)	+2.91 (C=O carbamate)	-	-
Butyl (**2**)	+2.20 (C=O lactone)	+2.92 (NH carbamate)	-	+2.97 (O carbamate)	-
Hexyl (**4**)	+2.22 (C=O lactone)	+2.98 (NH amide)	-	+2.61 (O carbamate)	-
Bn (**5**)	+2.89 (C=O lactone)	+2.98 (NH carbamate)	-	+3.01 (O carbamate)	-
4-BrBn (**6**)	+2.35 (C=O lactone)	+2.95 (NH carbamate)	+3.01 (C=O carbamate)	+3.32 (O carbamate)	-
4-NO_2_Bn (**7**)	+2.07 (C=O lactone)	+2.99 (NH carbamate)	-	+3.04 (O carbamate)	++ 2.22 and 3.05 (NO_2_)
ButylS (**9**)	+2.63 (C=O lactone)	+3.00 (NH carbamate)	+3.09 (C=O carbamate)	+3.19 (S carbamate)	-
BnS (**10**)	+2.89 (C=O lactone)	+2.98 (NH carbamate)	+3.13 (C=O carbamate)	+3.08 (S carbamate)	-
4-NO_2_BnS (**11**)	+2.12 (C=O lactone)	+3.14 (NH carbamate)	-	+3.25 (S carbamate)	++ 1.96 and 3.85 (NO_2_)
Hydrazide (**12**)	+2.45 (C=O lactone)	+2.94 (NH hydrazide)	-	+2.21 (C=O hydrazide)	-

## Supplementary Materials

The following are available online at https://www.mdpi.com/2218-273X/10/3/455/s1, ^1^H NMR and ^13^C NMR data.
