# Supplementary material for: Biological Evaluation and Docking Studies of New Carbamate, Thiocarbamate, and Hydrazide Analogues of Acyl Homoserine Lactones as Vibrio fischeri-Quorum Sensing Modulators
Source: Biomolecules. 2020 Mar 15;10(3):455. doi: 10.3390/biom10030455 (PMC7175117; doi:10.3390/biom10030455)
Supplement: Supplementary file 1 [file biomolecules-10-00455-s001.pdf]

Biological Evaluation and Docking Studies of New Carbamate, Thiocarbamate, and Hydrazide Analogues of Acyl Homoserine Lactones as *Vibrio fischeri*-Quorum Sensing Modulators

Qiang Zhang, Yves Queneau* and Laurent Soulère*

Univ Lyon, Université Claude Bernard Lyon 1, INSA Lyon, CPE Lyon, UMR 5246, CNRS, ICBMS, Institut de Chimie et de Biochimie Moléculaires et Supramoléculaires, Chimie Organique et Bioorganique, Bât. E. Lederer, 1 rue Victor Grignard F-69622 Villeurbanne, France.

Supplementary data – ^{1}H NMR and ^{13}C NMR spectra

---

* Corresponding authors at: ICBMS, Chimie Organique et Bioorganique, Bât. E. Lederer, 1 rue Victor Grignard F-69622 Villeurbanne, France.
E-mail addresses: yves.queneau@insa-lyon.fr and laurent.soulere@insa-lyon.fr

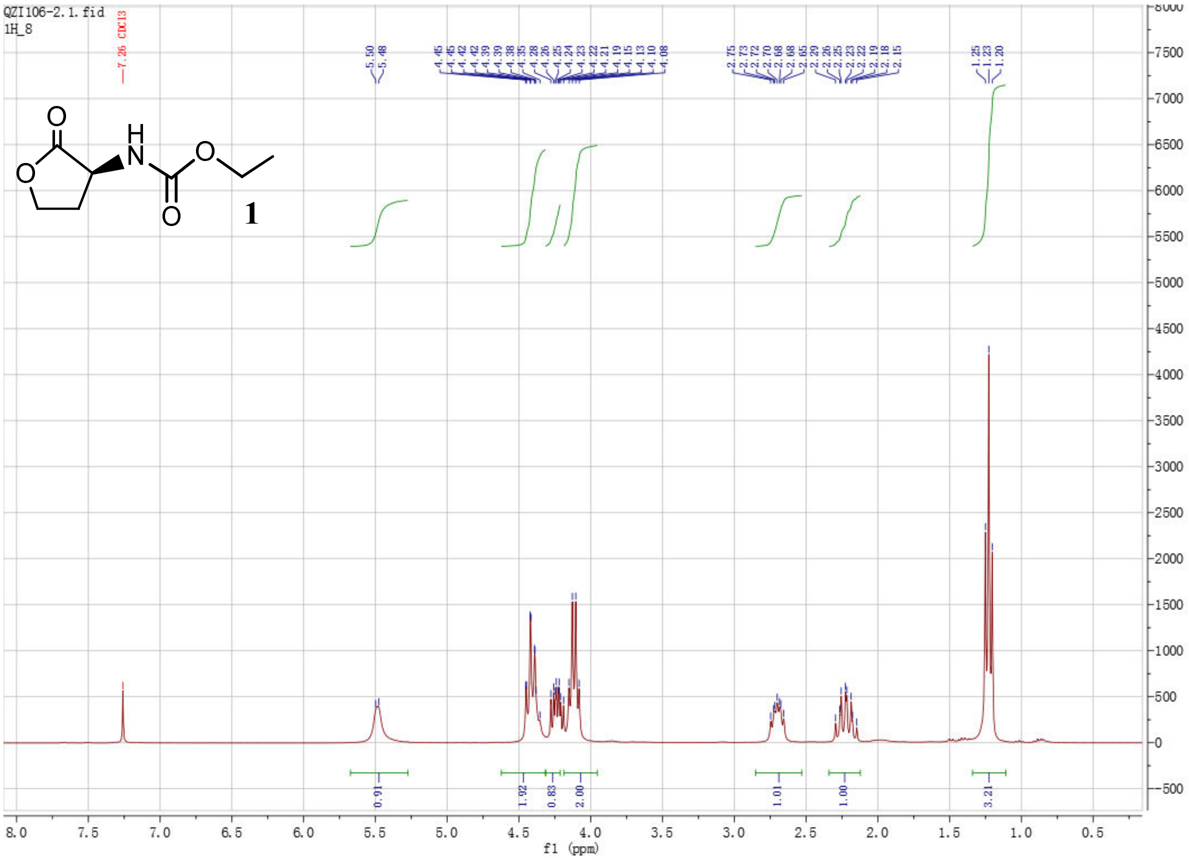
QZI106-2.1.fid
1H_8
7.26 CDCl3
1
f1 (ppm)

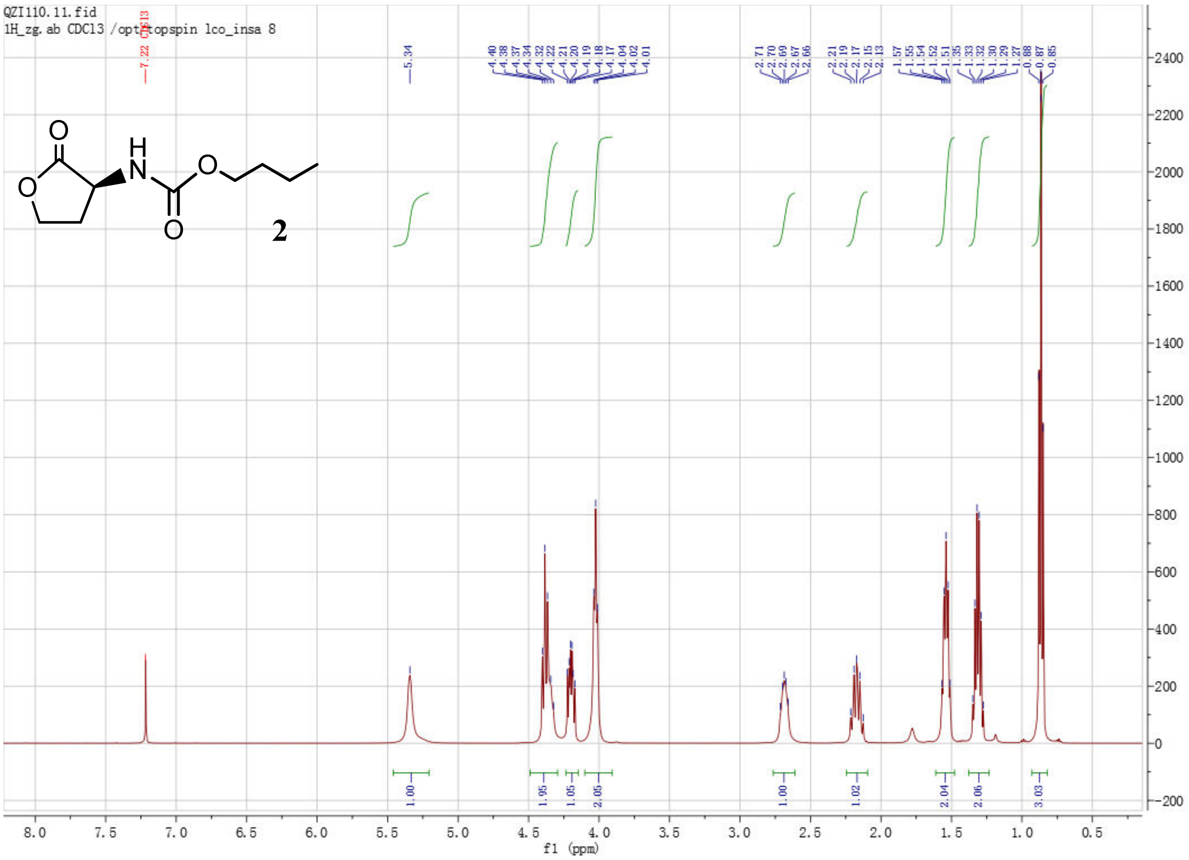
QZI110.11.fid
1H_zg.ab CDCl3 /opt/topspin lco_insa 8
7.22 CDCl3
2
f1 (ppm)

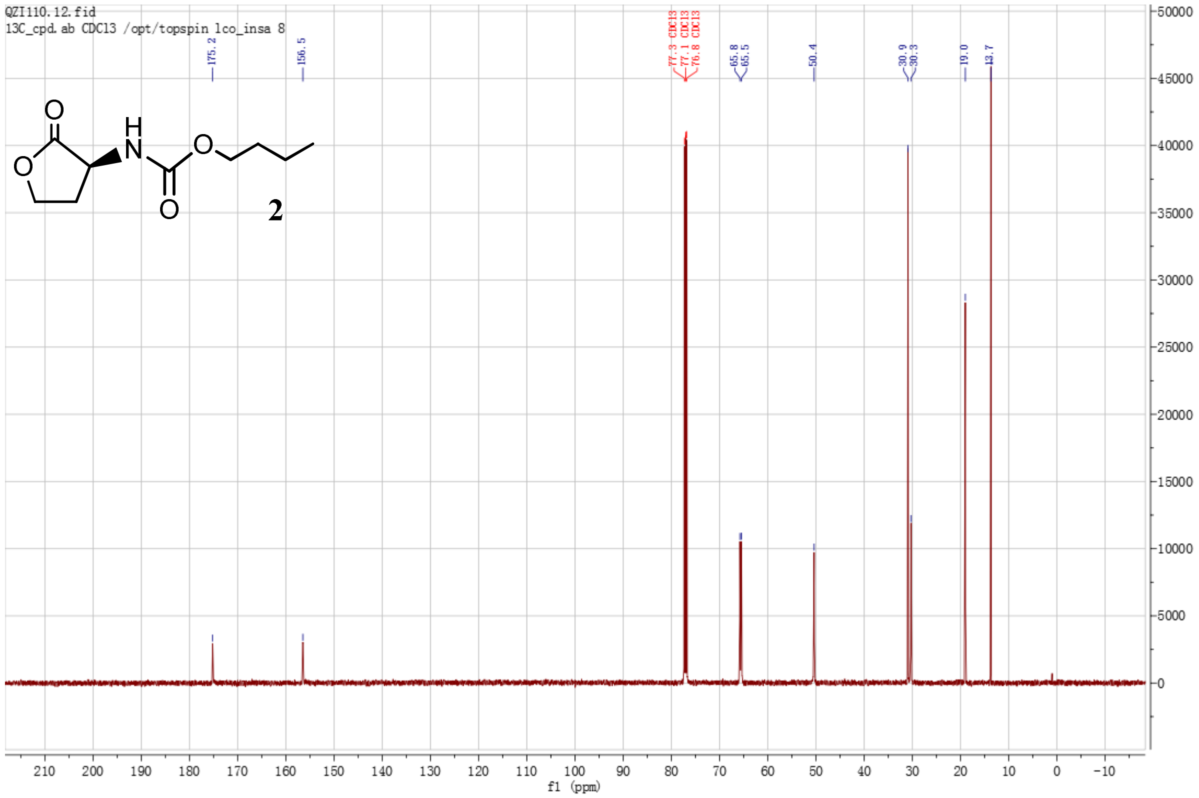
QZI110.12.fid
13C_cpd.ab CDCl3 /opt/topspin lco_insa 8
175.2
156.5
77.3 CDCl3
77.1 CDCl3
76.8 CDCl3
65.8
65.5
50.4
30.9
30.3
19.0
13.7
2
f1 (ppm)

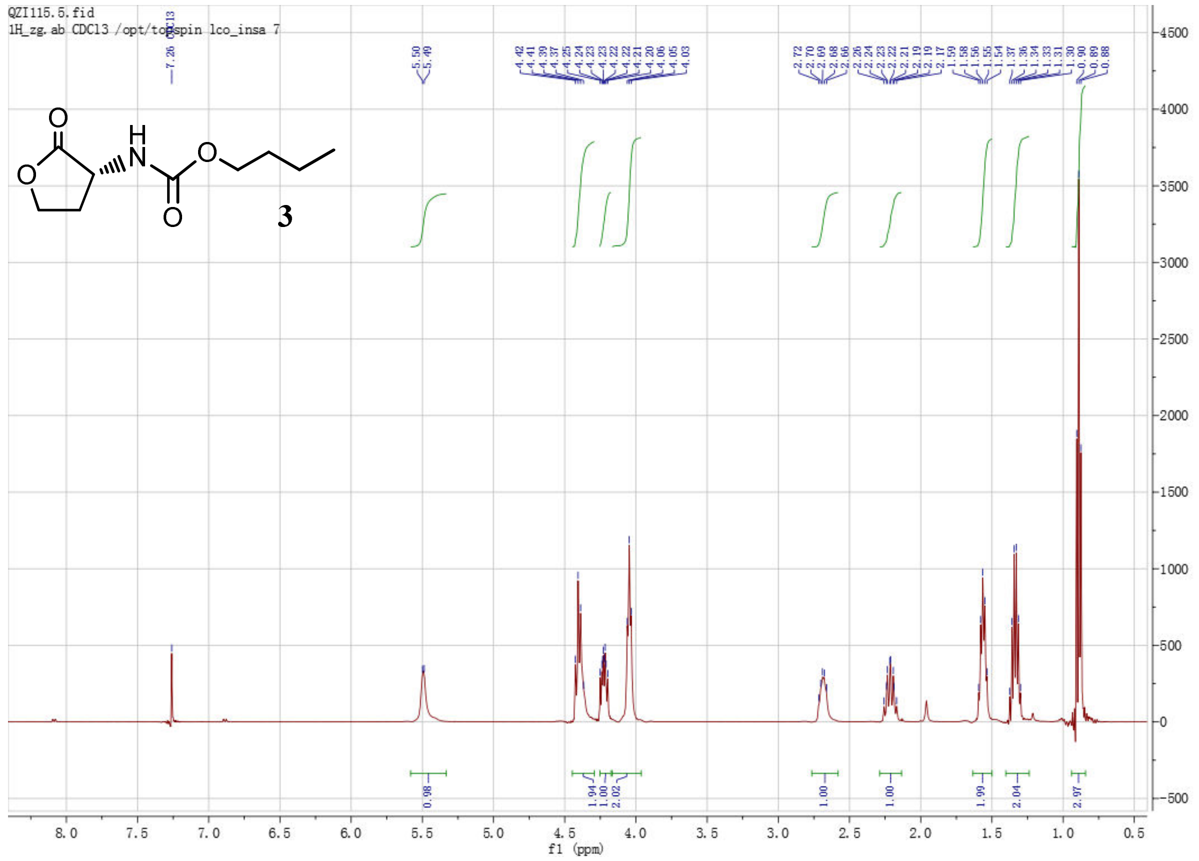
QZI115.5.fid
1H_zg.ab CDCl3 /opt/topspin lco_insa 7
7.26 CDCl3
5.50
5.49
0.98
1.94
1.00
2.02
1.00
1.00
1.99
2.04
2.97
3
f1 (ppm)

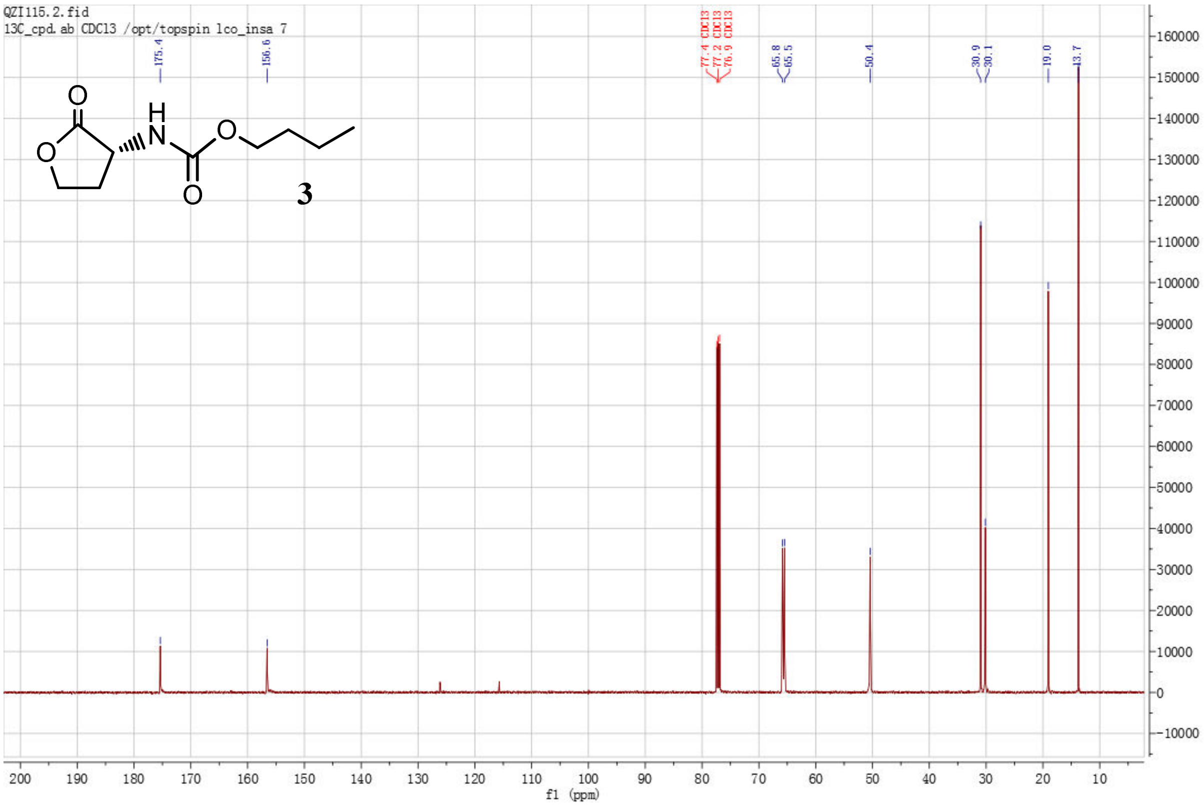
QZI115.2.fid
13C_cpd.ab CDCl3 /opt/topspin lco_insa 7
175.4
156.6
77.4 CDCl3
77.2 CDCl3
76.9 CDCl3
65.8
65.5
50.4
30.9
30.1
19.0
13.7
3
f1 (ppm)

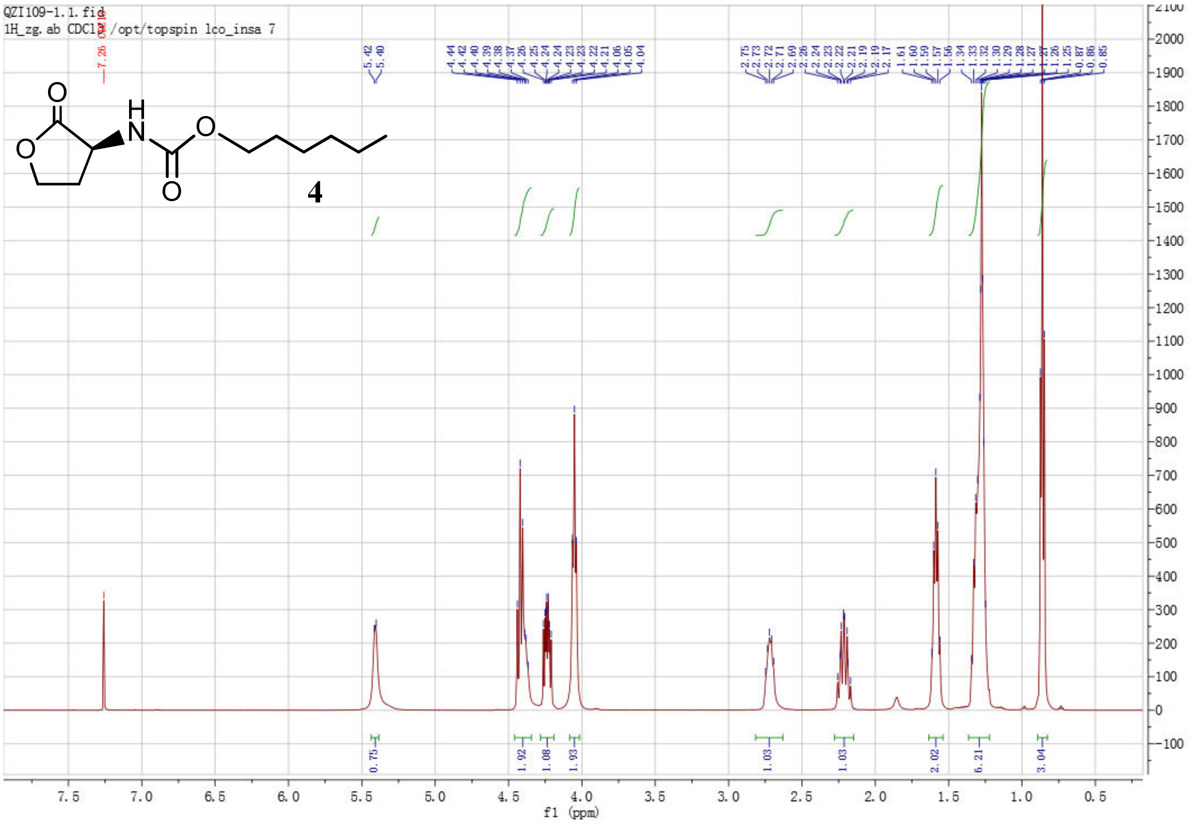
QZI109-1.1.fid
1H_zg.ab CDCl3 /opt/topspin lco_insa 7
7.26
5.42
5.40
4
0.75
1.92
1.08
1.93
1.03
1.03
2.02
6.21
3.04
f1 (ppm)

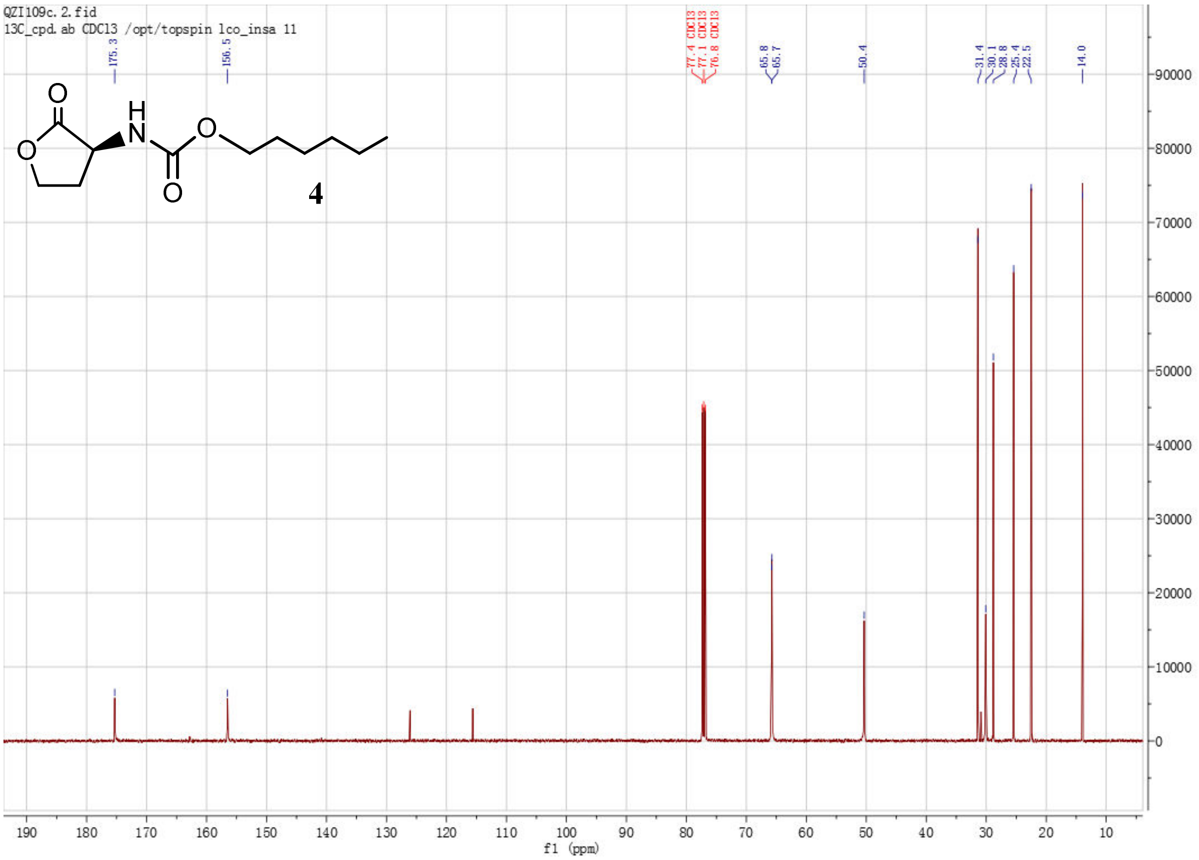
QZI109c.2.fid
13C_cpd.ab CDCl3 /opt/topspin lco_insa 11
175.3
156.5
77.4 CDCl3
77.1 CDCl3
76.8 CDCl3
65.8
65.7
50.4
31.4
30.1
28.8
25.4
22.5
14.0
4
f1 (ppm)

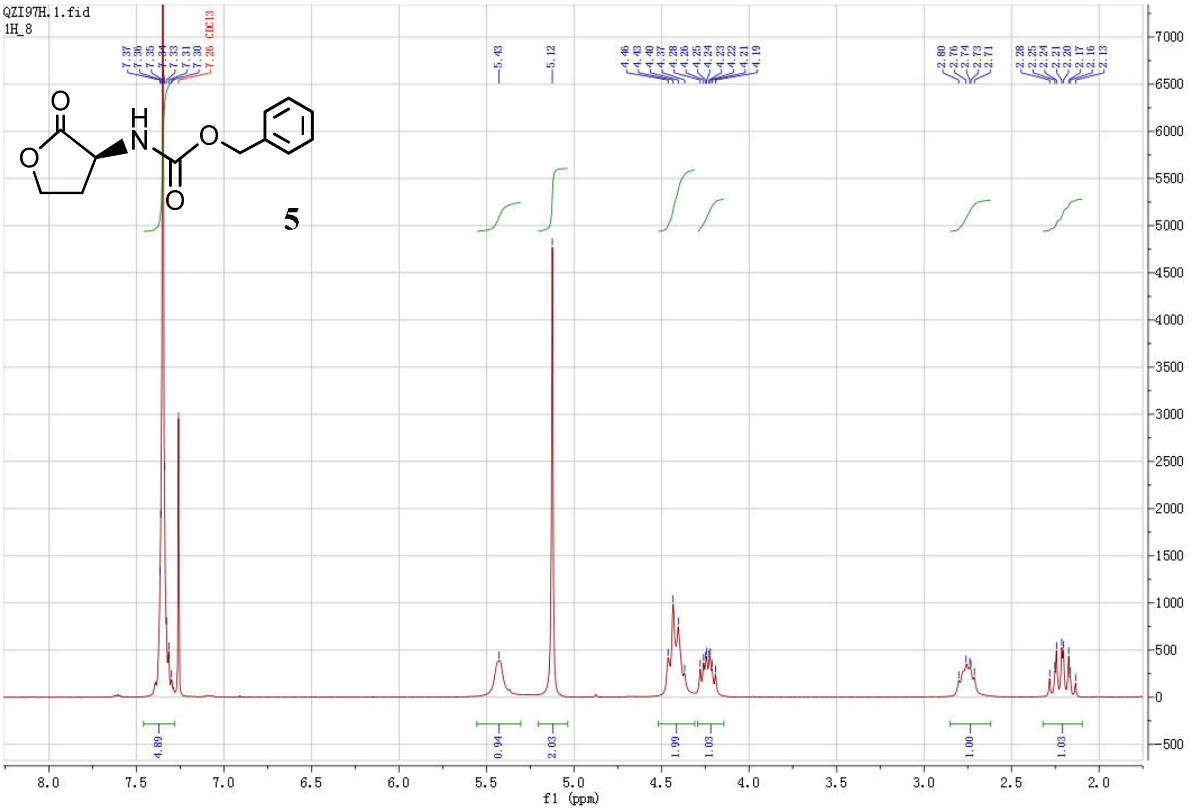
QZI97H.1.fid
1H_8
7.37
7.36
7.35
7.34
7.33
7.31
7.30
7.26 CDCl3
5.43
5.12
4.46
4.43
4.40
4.37
4.28
4.26
4.25
4.24
4.23
4.22
4.21
4.19
2.80
2.76
2.74
2.73
2.71
2.28
2.25
2.24
2.21
2.20
2.17
2.16
2.13
5
4.89
0.94
2.03
1.99
1.03
1.00
1.03
f1 (ppm)

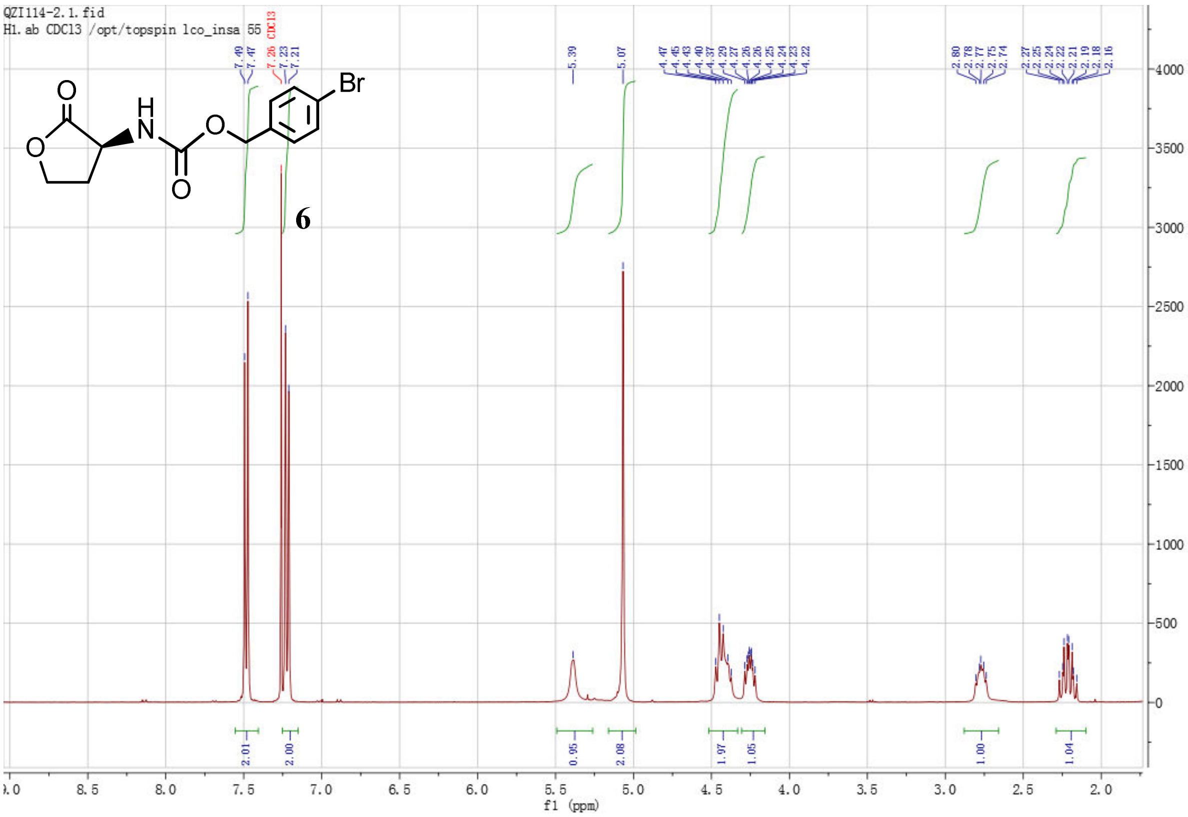
QZI114-2.1.fid
H1.ab CDCl3 /opt/topspin lco_insa 55
7.49
7.47
7.26 CDCl3
7.23
7.21
5.39
5.07
4.47
4.45
4.43
4.40
4.37
4.29
4.27
4.26
4.26
4.25
4.24
4.23
4.22
2.80
2.78
2.77
2.75
2.74
2.27
2.25
2.24
2.22
2.21
2.19
2.18
2.16
Br
O
H
N
O
O
O
6
2.01
2.00
0.95
2.08
1.97
1.05
1.00
1.04
f1 (ppm)

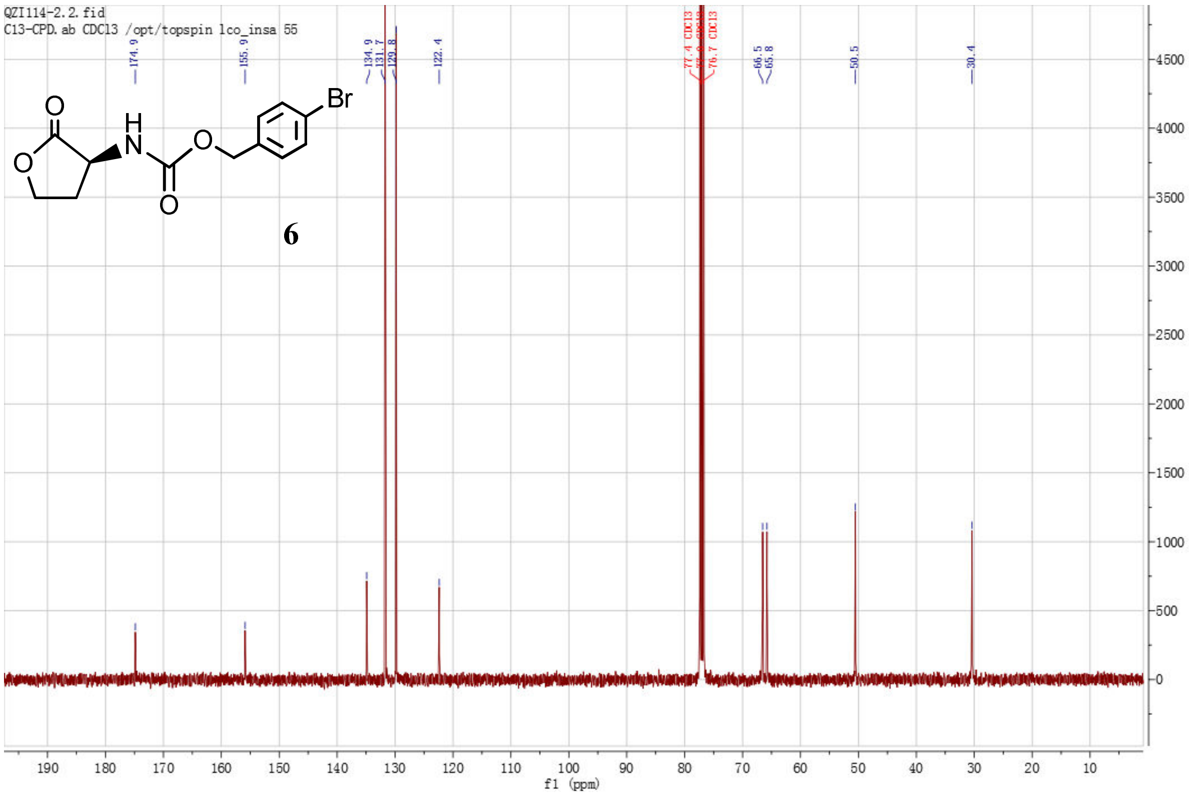
QZI114-2.2.fid
C13-CPD.ab CDCl3 /opt/topspin lco_insa 55
174.9
155.9
134.9
131.7
129.8
122.4
77.4 CDCl3
76.7 CDCl3
66.5
65.8
50.5
30.4
Br
O
H
N
O
O
O
6
f1 (ppm)

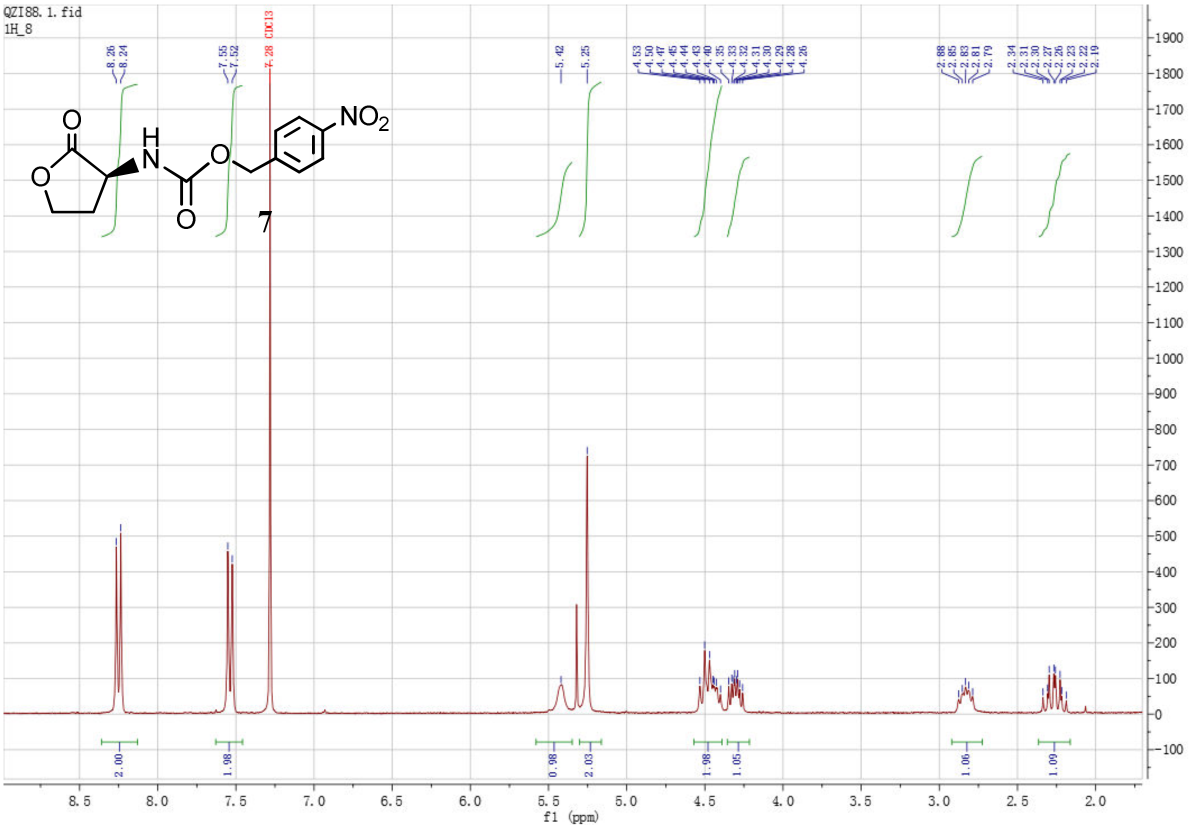
QZI88.1.fid
1H_8
NO2
7
f1 (ppm)

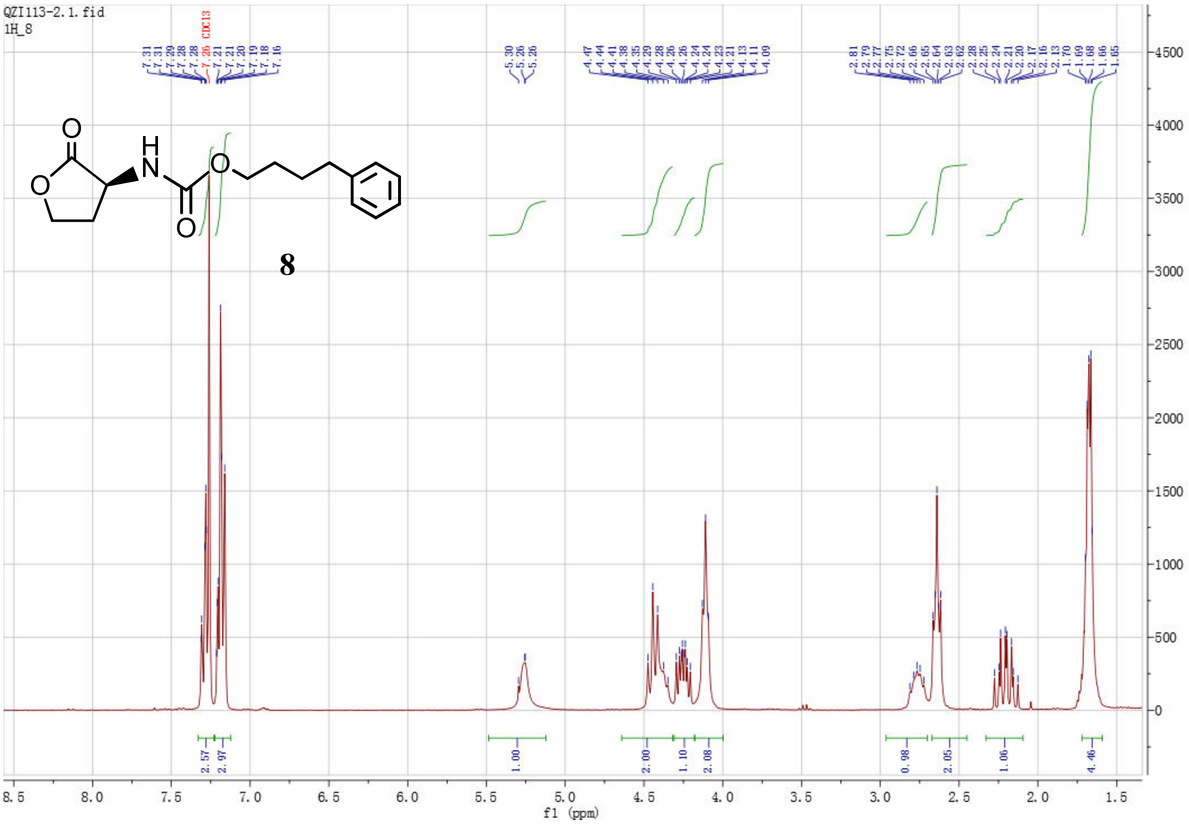
QZI113-2.1.fid
1H_8
8
f1 (ppm)

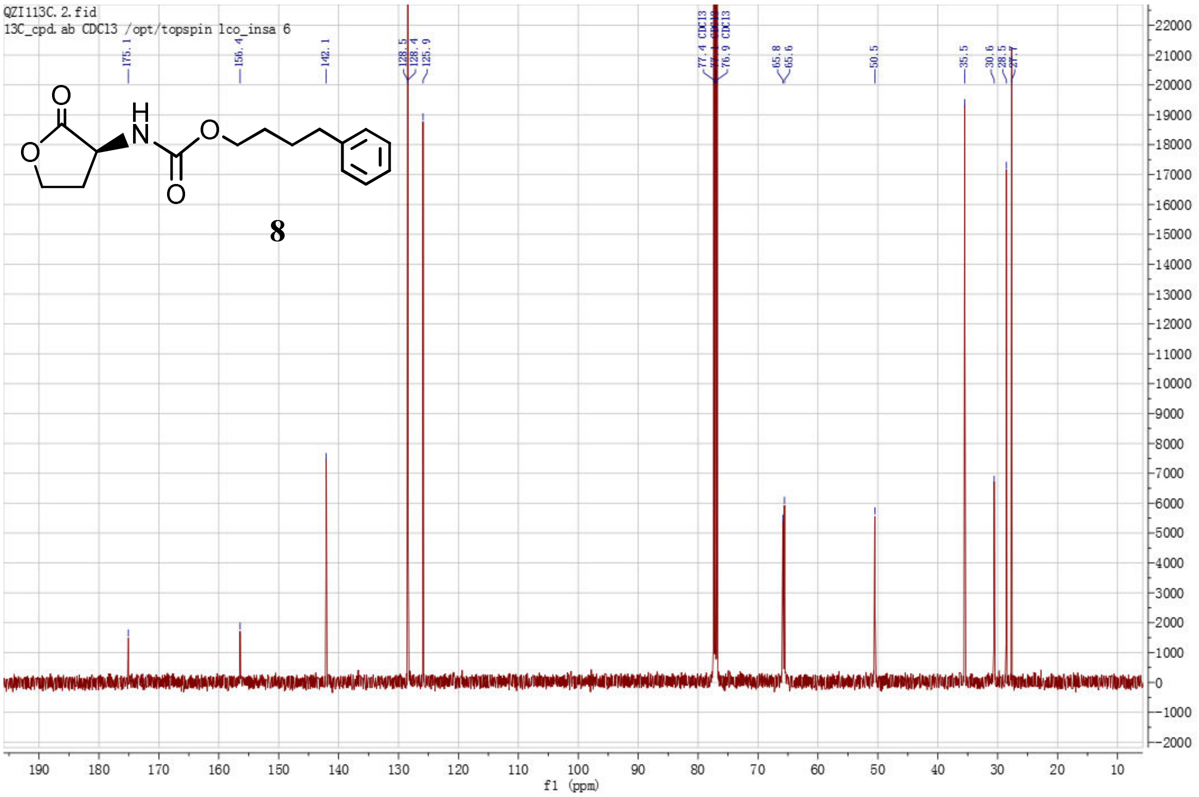
QZI113C.2.fid
13C_cpd.ab CDCl3 /opt/topspin lco_insa 6
175.1
156.4
142.1
128.5
128.4
125.9
77.4 CDCl3
76.9 CDCl3
65.8
65.6
50.5
35.5
30.6
28.5
8
f1 (ppm)

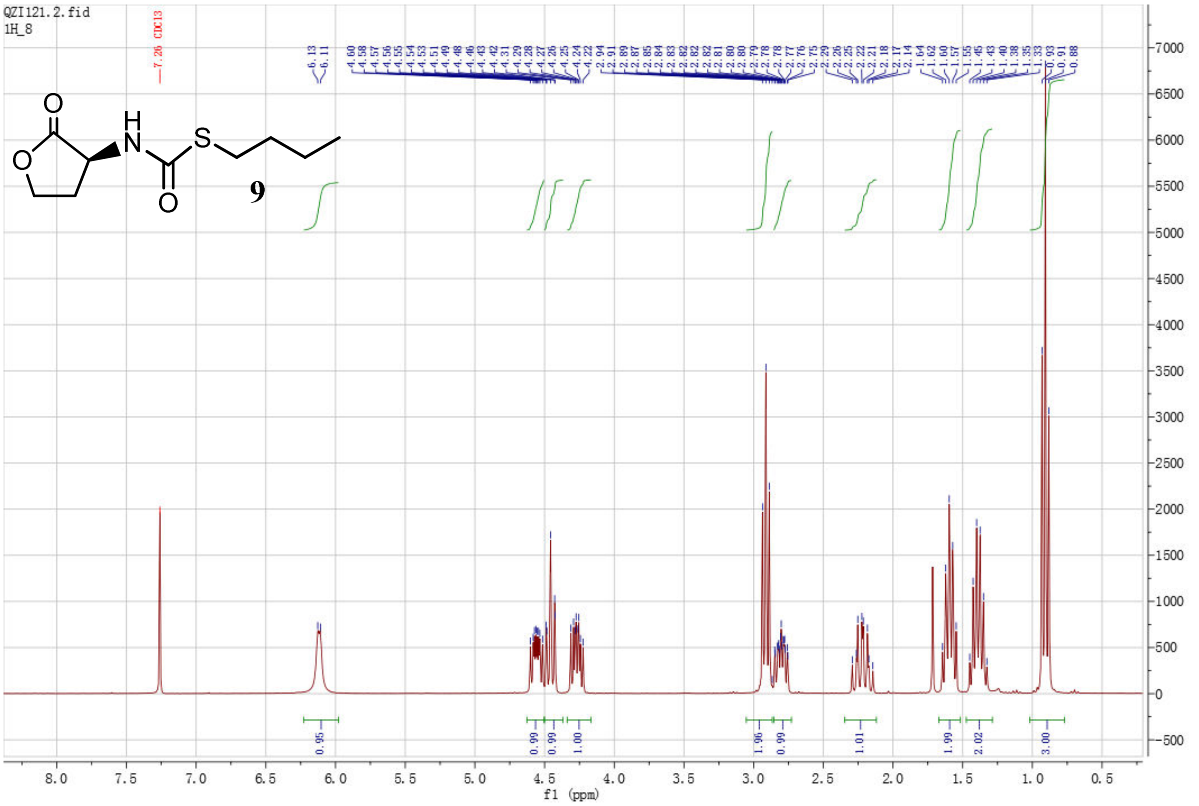
QZI121.2.fid
1H_8
7.26 CDCl3
6.13
6.11
9
0.95
0.99
0.99
1.00
1.96
0.99
1.01
1.99
2.02
3.00
f1 (ppm)

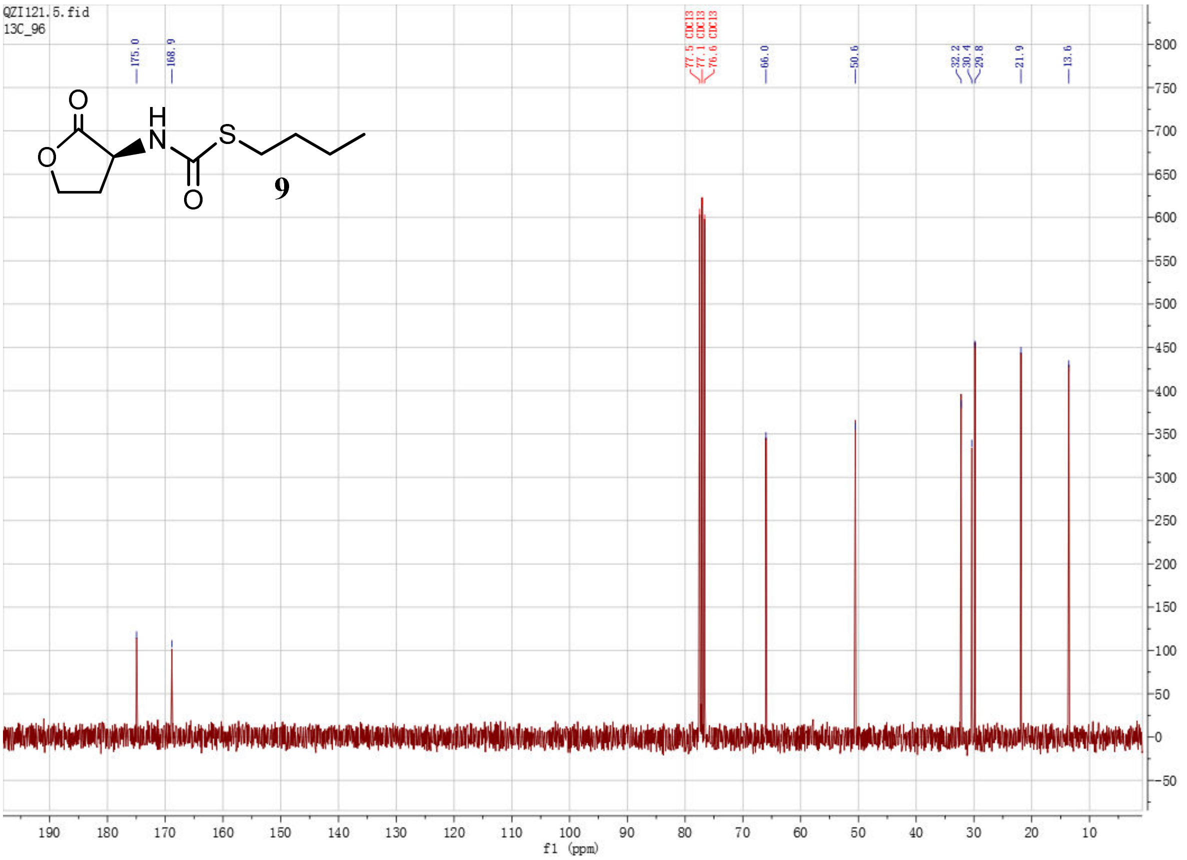
QZI121.5.fid
13C_96
175.0
168.9
77.5 CDCl3
77.1 CDCl3
76.6 CDCl3
66.0
50.6
32.2
30.4
29.8
21.9
13.6
f1 (ppm)

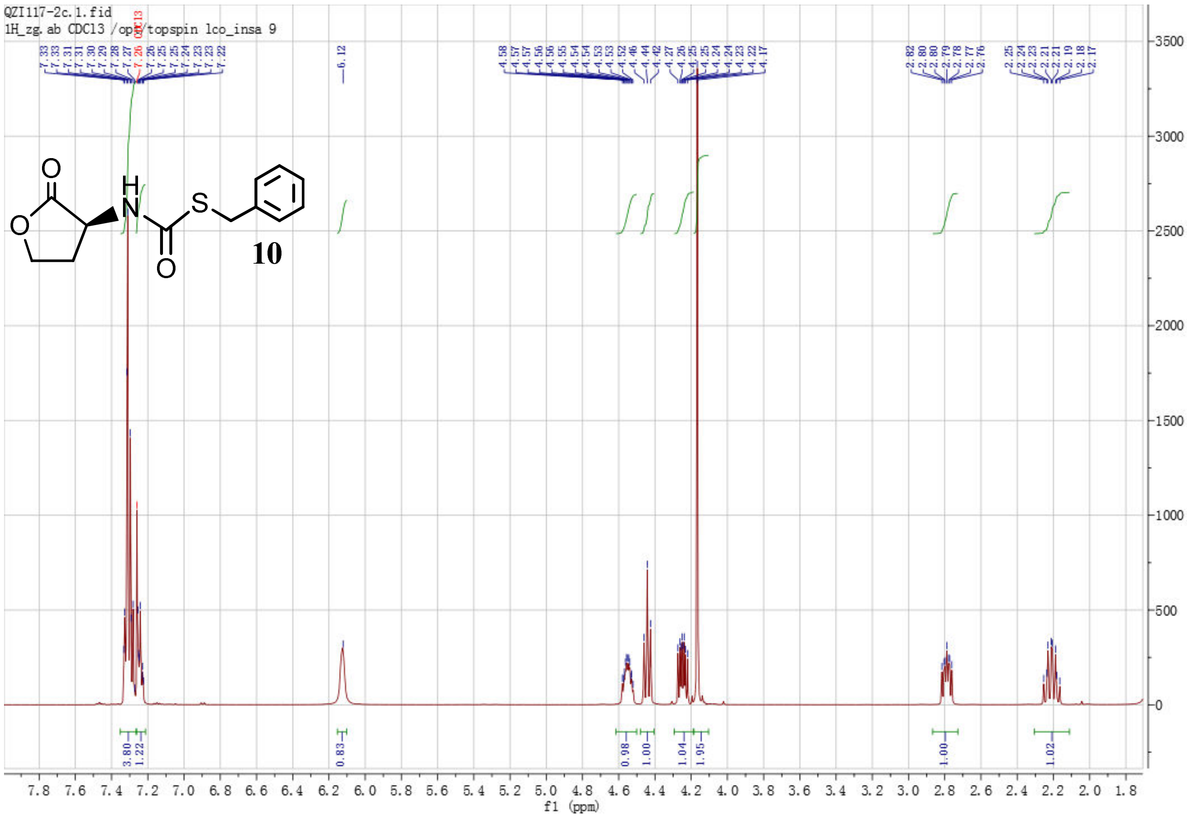
QZI117-2c.1.fid
1H_zg.ab CDCl3 /opt/topspin lco_insa 9
CDCl3
6.12
f1 (ppm)

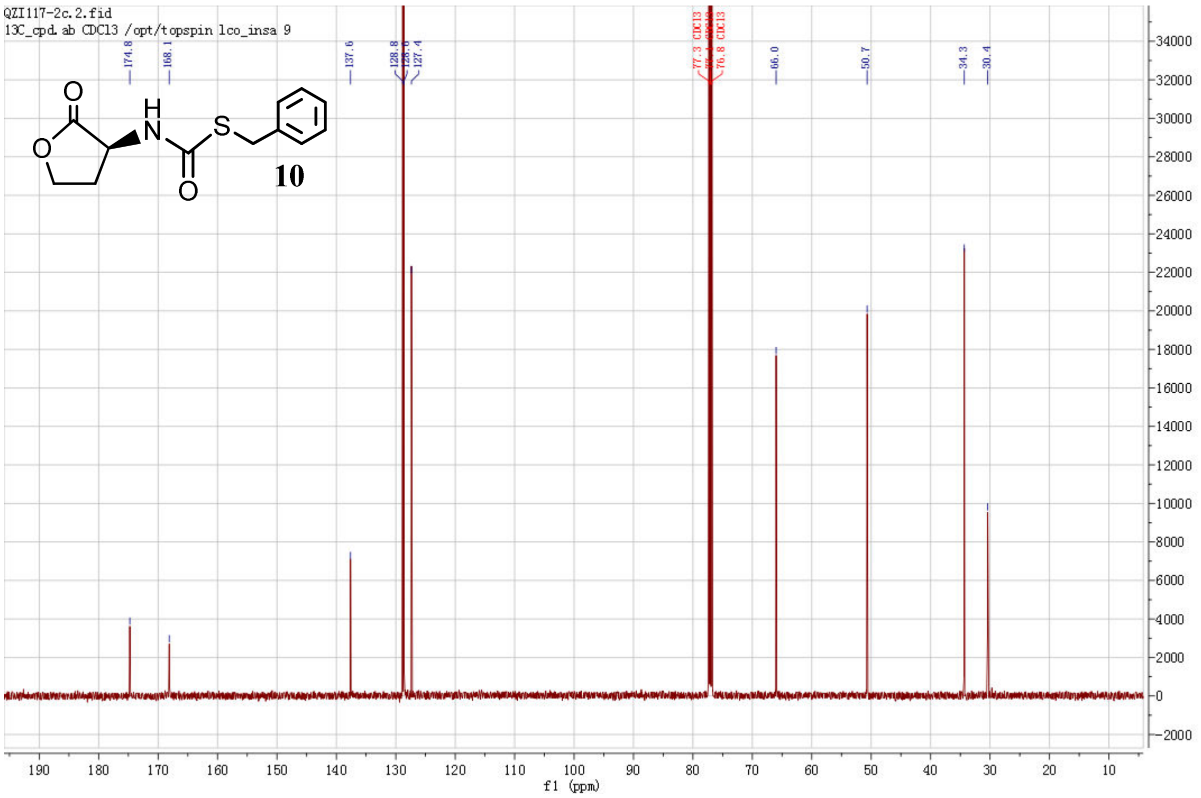
QZI117-2c.2.fid
13C_cpd.ab CDCl3 /opt/topspin lco_insa 9
174.8
168.1
137.6
128.8
127.4
77.3 CDCl3
76.8 CDCl3
66.0
50.7
34.3
30.4
10
f1 (ppm)

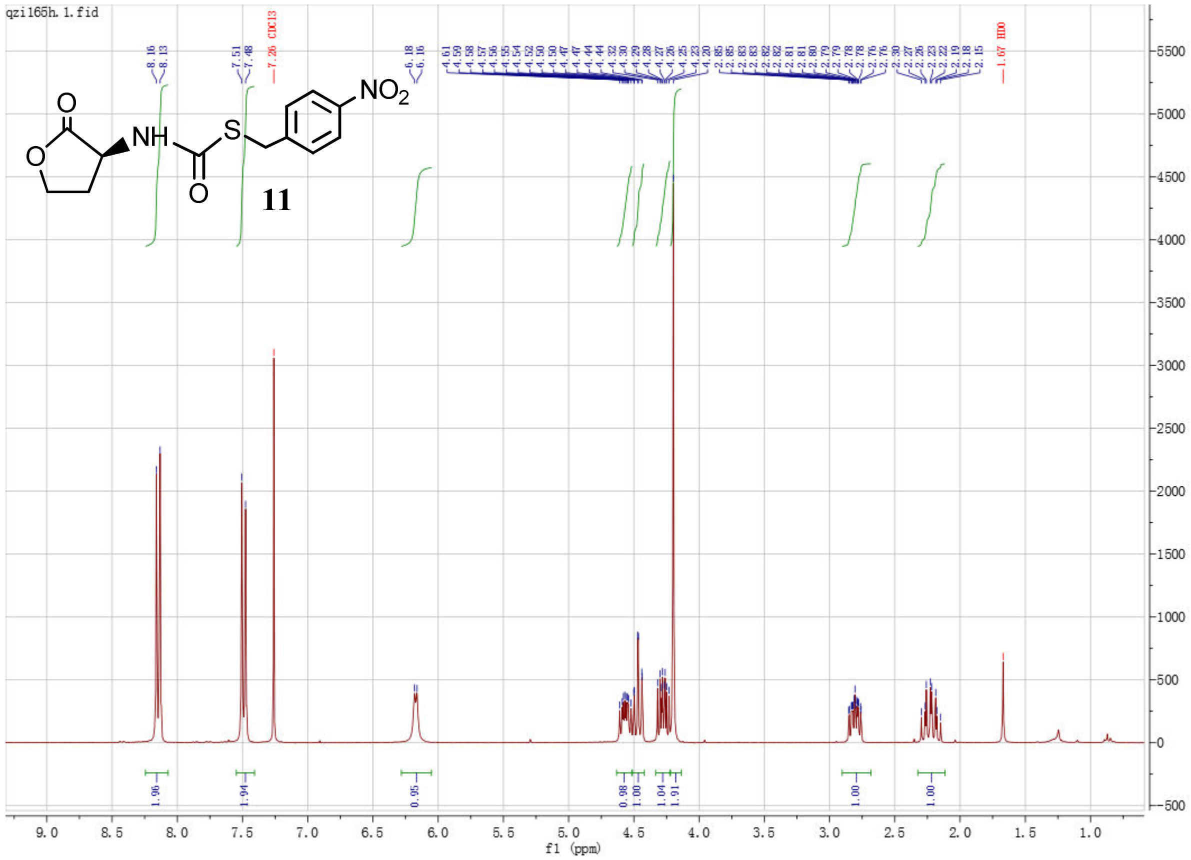
qzi165h.1.fid
8.16
8.13
7.51
7.48
7.26 CDCl3
6.18
6.16
1.67 HDO
11
1.96
1.94
0.95
0.98
1.00
1.04
1.91
1.00
1.00
f1 (ppm)

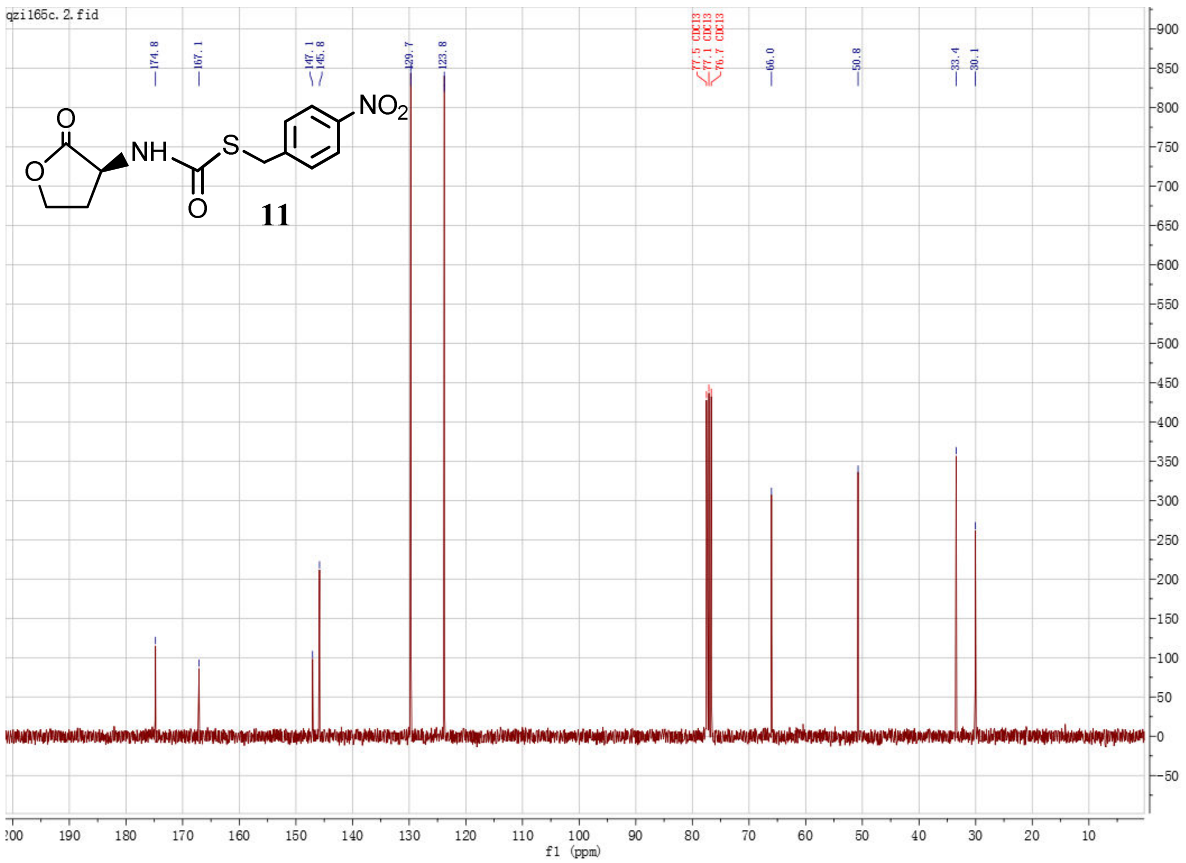
qzi165c.2.fid
174.8
167.1
147.1
145.8
129.7
123.8
77.5 CDCl3
77.1 CDCl3
76.7 CDCl3
66.0
50.8
33.4
30.1
NH
S
NO2
O
11
f1 (ppm)

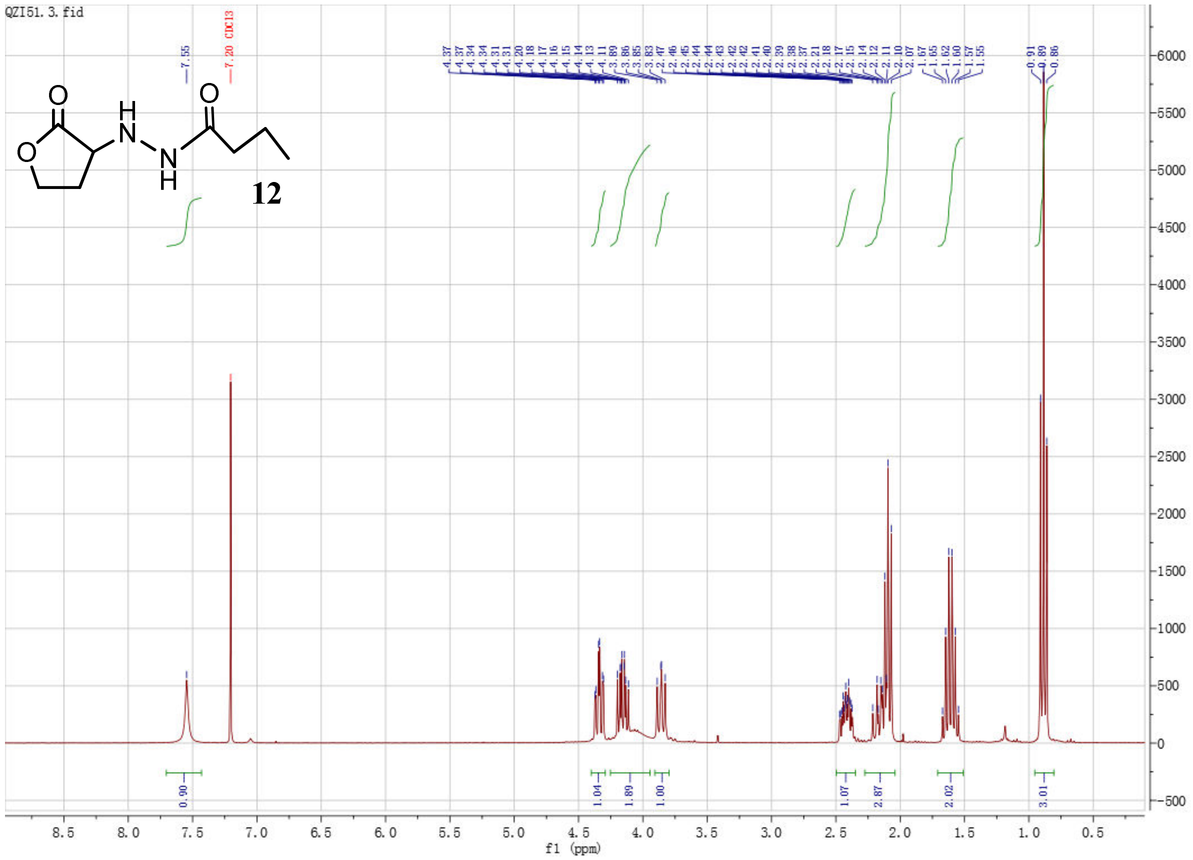
QZI51.3.fid
7.55
7.20 CDCl3
H
N
O
12
0.90
1.04
1.89
1.00
1.07
2.87
2.02
3.01
f1 (ppm)

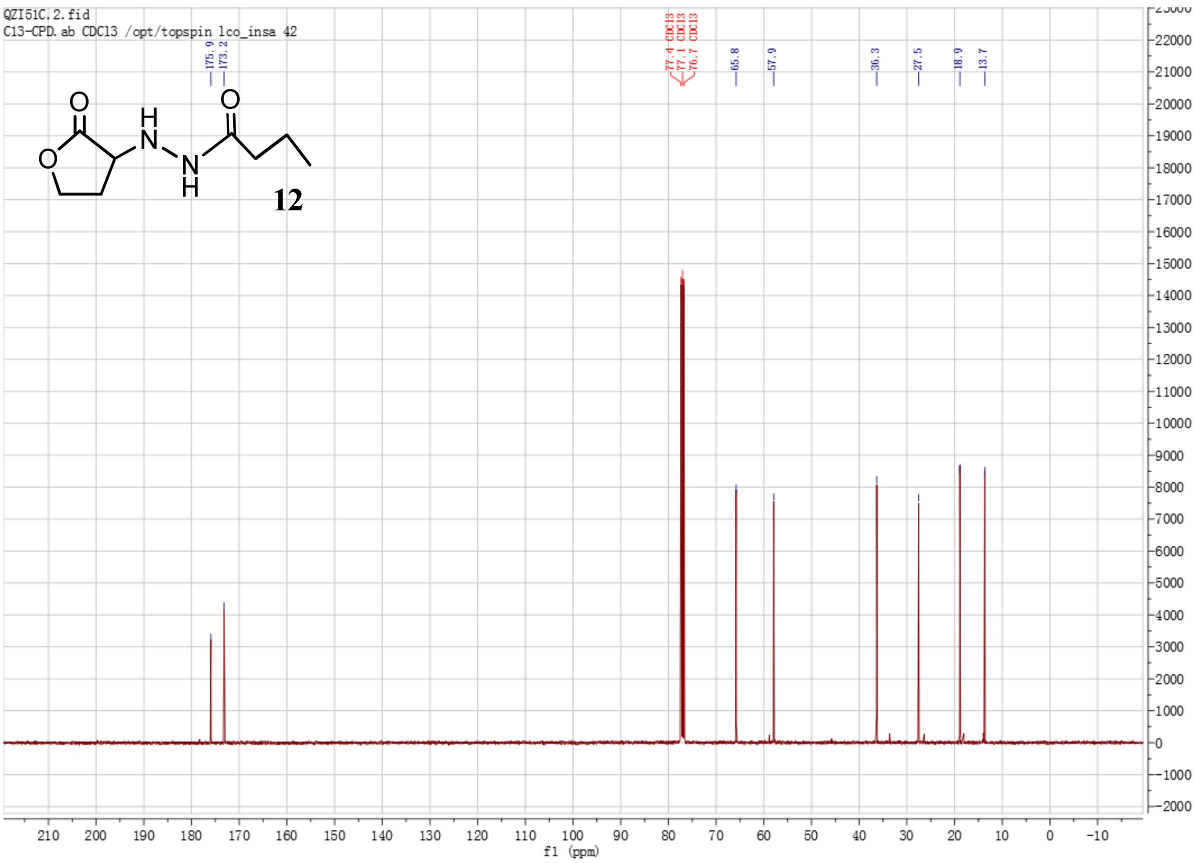
QZI51C.2.fid
C13-CPD.ab CDCl3 /opt/topspin lco_insa 42
175.9
173.2
77.4 CDCl3
77.1 CDCl3
76.7 CDCl3
65.8
57.9
36.3
27.5
18.9
13.7
12
f1 (ppm)

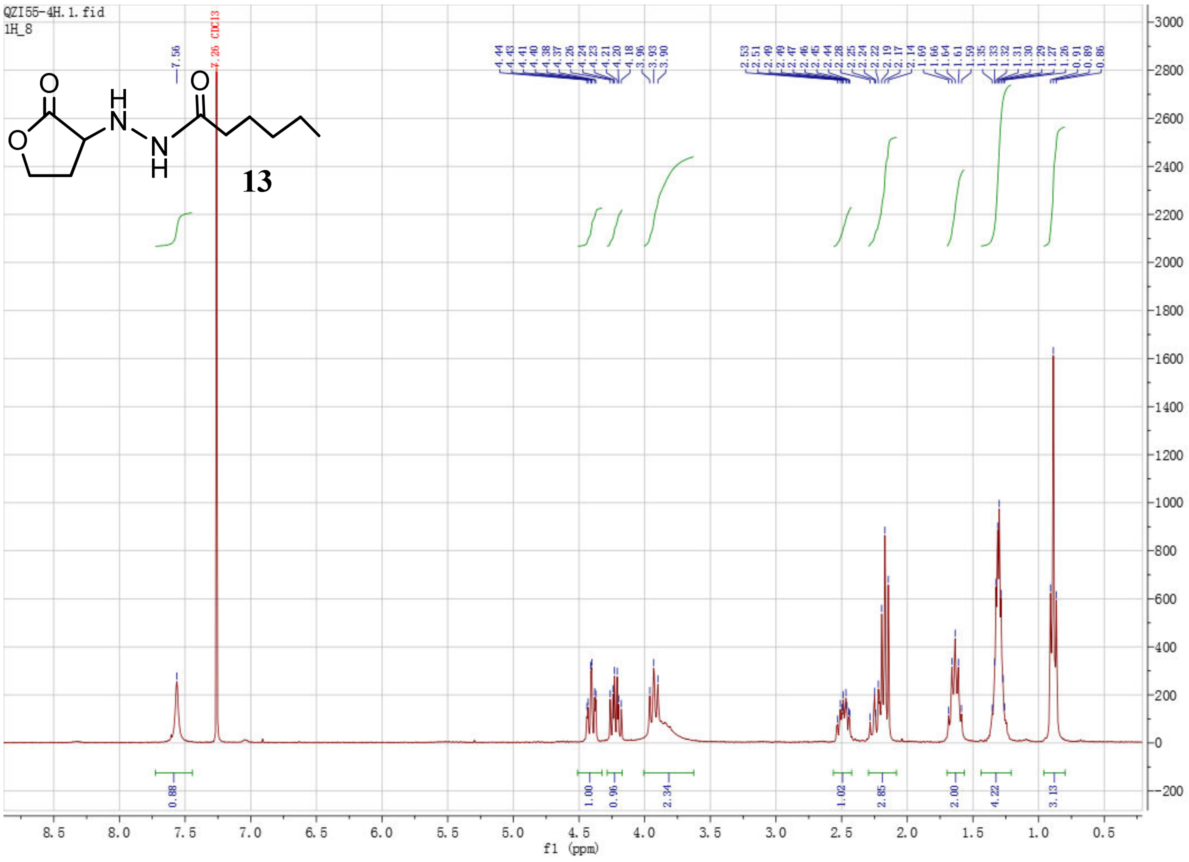
QZI55-4H.1.fid
1H_8
7.56
7.26 CDCl3
4.44 4.43 4.41 4.40 4.38 4.37 4.26 4.24 4.23 4.21 4.20 4.18 3.96 3.93 3.90
2.53 2.51 2.49 2.48 2.47 2.46 2.45 2.44 2.28 2.25 2.24 2.22 2.19 2.17 2.14 1.69 1.66 1.64 1.61 1.59 1.35 1.33 1.32 1.31 1.30 1.29 1.27 1.26 0.91 0.89 0.86
13
0.88
1.00
0.96
2.34
1.02
2.85
2.00
4.22
3.13
f1 (ppm)

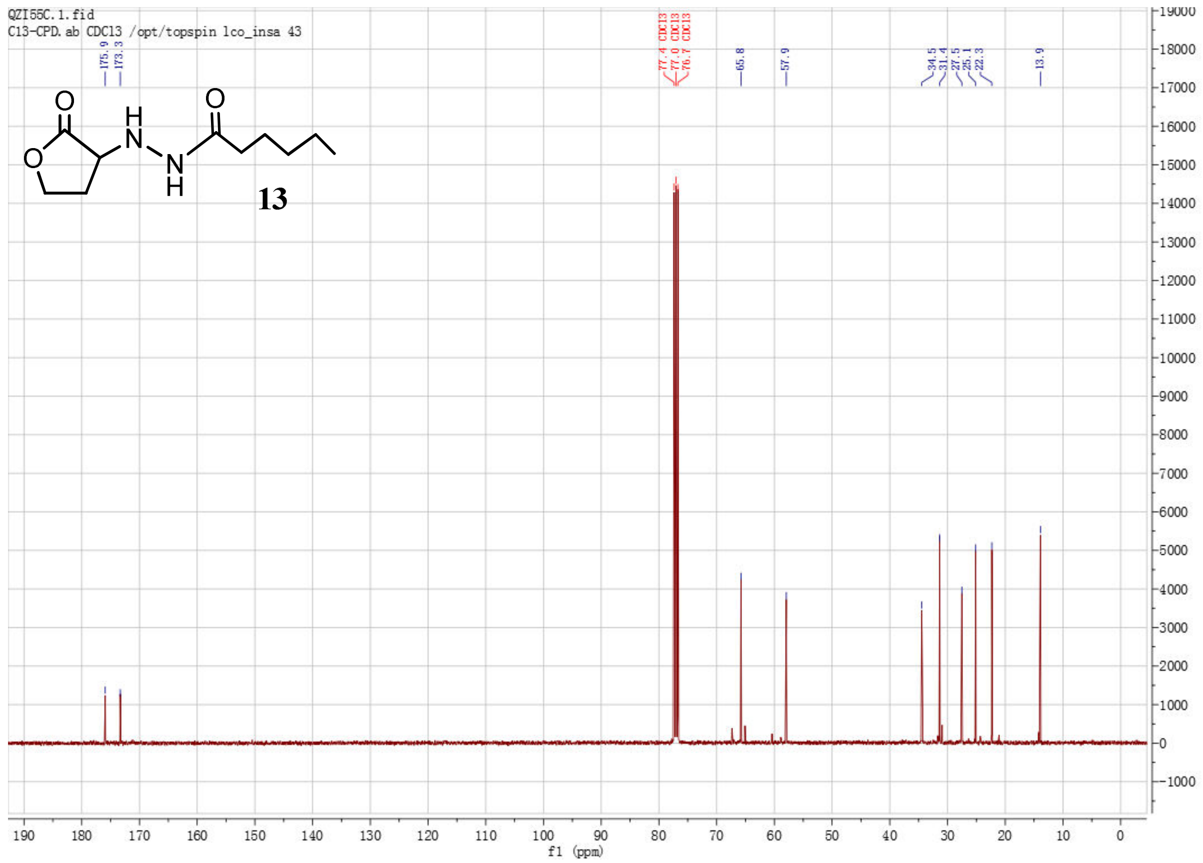
QZI55C.1.fid
C13-CPD.ab CDCl3 /opt/topspin lco_insa 43
175.9
173.3
77.4 CDCl3
77.0 CDCl3
76.7 CDCl3
65.8
57.9
34.5
31.4
27.5
25.1
22.3
13.9
13
f1 (ppm)

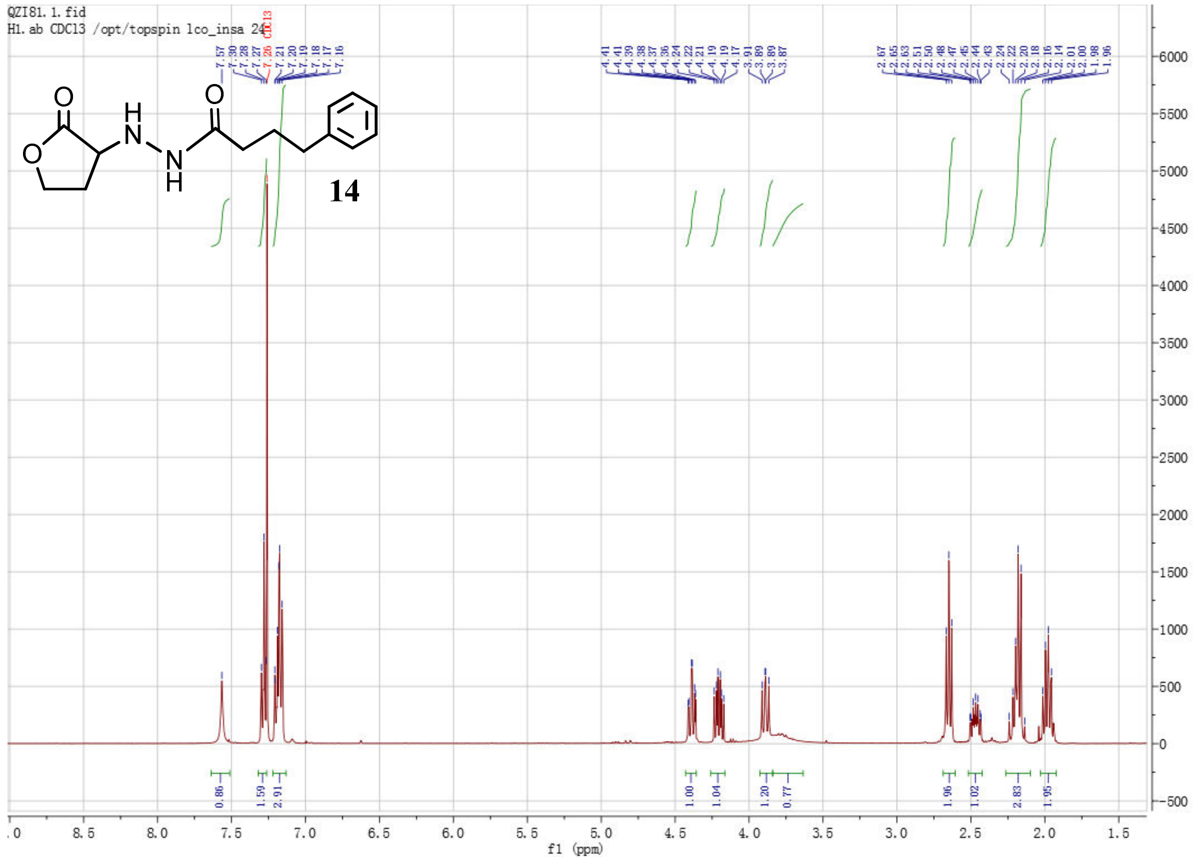
QZI81.1.fid
H1.ab CDCl3 /opt/topspin lco_insa 24
14
0.86
1.59
2.91
1.00
1.04
1.20
0.77
1.96
1.02
2.83
1.95
f1 (ppm)

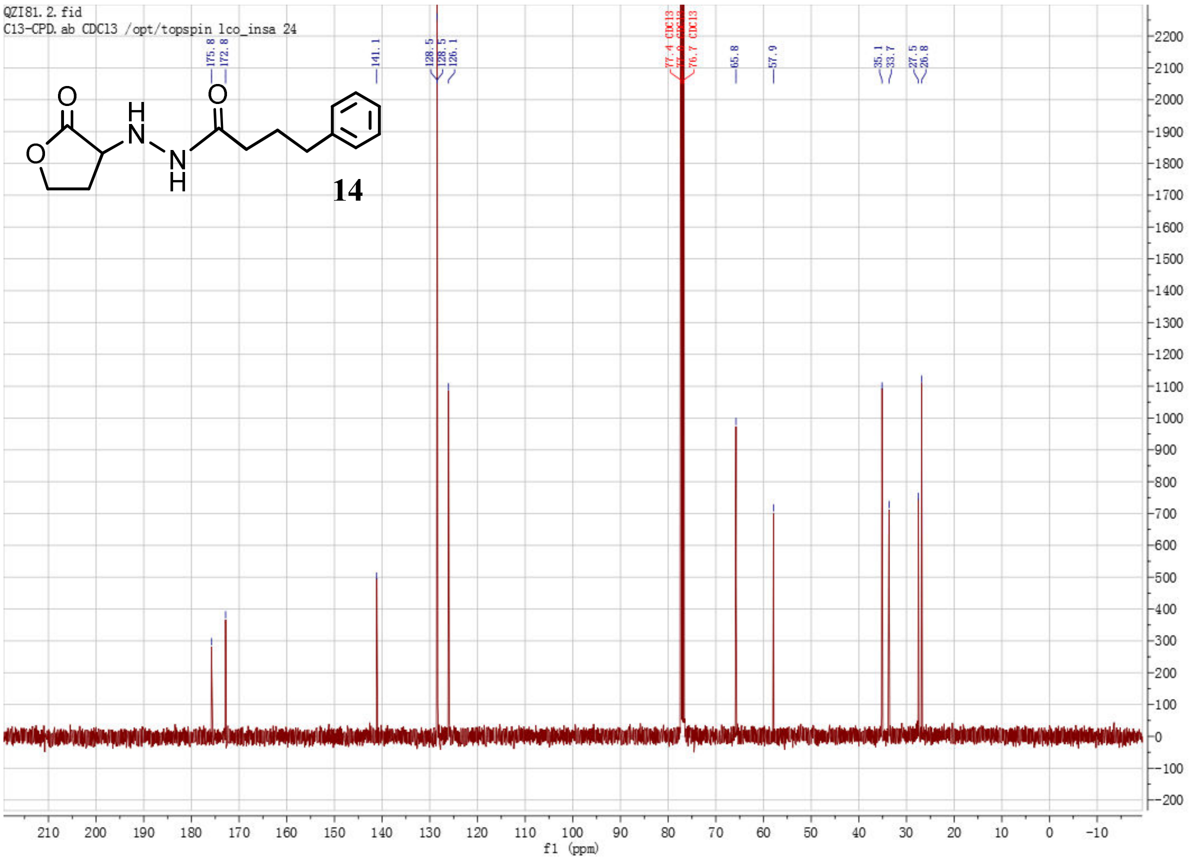
QZI81.2.fid
C13-CPD.ab CDCl3 /opt/topspin lco_insa 24
175.8
172.8
141.1
128.5
128.5
126.1
77.4 CDCl3
76.7 CDCl3
65.8
57.9
35.1
33.7
27.5
26.8
14
f1 (ppm)
